# Integrative Insights Into DYRK1A From Molecular Function to Therapeutic Advancement

**DOI:** 10.1111/cbdd.70366

**Published:** 2026-07-23

**Authors:** Sampriti Paul, Sonal Dubey, Prashant Tiwari

**Affiliations:** ^1^ College of Pharmaceutical Sciences Dayananda Sagar University Bengaluru South India

**Keywords:** Alzheimer disease, Down syndrome, DYRK1A, protein kinase inhibitor, structure–activity relationship

## Abstract

Dual‐specificity tyrosine‐phosphorylation‐regulated kinase 1A (DYRK1A), located within the Down syndrome critical region and implicated in Alzheimer's disease (AD), Parkinson's disease (PD), and context‐dependent cancer biology, represents a high‐value yet challenging therapeutic target. This review compiles comprehensive structure–activity relationship (SAR) insights essential for medicinal chemists designing selective DYRK1A inhibitors. We detail the molecular architecture of the ATP‐binding pocket of DYRK1A, key regulatory residues (Lys188, Phe238, Glu239, Leu241), and structure–function relationships governing inhibitor classes: ATP‐competitive agents, ATP‐non‐competitive inhibitors, and Proteolysis‐Targeting Chimeras (PROTAC) degraders with emphasis on functional group modifications and scaffold optimization strategies. Readers will gain actionable insights on binding mode predictions, potency‐selectivity trade‐offs, and prioritization of lead compounds for preclinical validation. The framework addresses pharmacokinetic property optimization and selectivity profiling across kinase families, enabling researchers to accelerate rational inhibitor design and facilitate translation of DYRK1A therapeutics into clinical trials for neurodegenerative and developmental disorders.

## Graphical abstract

At the center the 3D structure of DYRK1A has been displayed. The dual‐function of DYRK1A involved in cancer and neurodegeneration, i.e., its overexpression drives Down syndrome, neurodegenerative disease etc., and underexpression causes intellectual disability, seizures, etc. DYRK1A, in cancer acts context‐dependent kinase, either functions as oncogenic or tumor suppressor. The therapeutic strategies including the enzyme inhibitor targeting the kinase active site alongside gene therapy approaches for managing disease pathology.

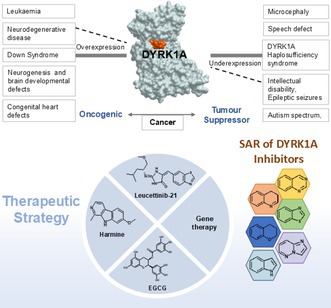

**Abbreviations**
AAV: adeno‐associated virus
AD: Alzheimer's disease
ADME: absorption, distribution, metabolism, excretion
Akt: protein kinase B
APP: amyloid precursor protein
ASK1: apoptosis signal‐regulating kinase 1
ATP: adenosine triphosphate
Aβ: amyloid‐beta peptide
BBB: blood–brain barrier
BRCA1: breast cancer type 1 susceptibility protein
CBP: CREB‐binding protein
CDK: cyclin‐dependent kinase
CHD: congenital heart defects
CHD2: chromodomain‐Helicase‐DNA‐binding protein 2
CLK: CDC‐like kinase
c‐MET: MET proto‐oncogene receptor
CMGC: CDK, MAPK, GSK3, CLK kinase group
CNS: central nervous system
CREB: cAMP response element binding protein
CRISPR: clustered regularly interspaced short palindromic repeats
CTD: C‐terminal domain
DDR: DNA damage response
DFG: aspartate‐phenylalanine‐glycine motif
DH: DYRK homology
DREAM: DP, RB‐like, E2F and MuvB complex
DREAM complex: dimerization partner, RB‐like, E2F and Multi‐vulval class B complex
DS: Down Syndrome
DSCR: Down Syndrome Critical Region
DYRK1A: dual‐specificity Tyrosine‐phosphorylation‐Regulated Kinase 1A
E2F1: E2F transcription factor 1
EGFR: epidermal growth factor receptor
EMT: epithelial‐to‐mesenchymal transition
ERK: extracellular signal‐regulated kinase
FOXO1: Forkhead Box O1
GLI1: GLI family zinc finger 1
GSK‐3β: glycogen synthase kinase 3 beta
H3S57: histone H3 Serine 57
H3T45: histone H3 Threonine 45
HATs: histone acetyltransferases
HP1: heterochromatin protein 1
iPSC: induced pluripotent stem cell
JNK: c‐Jun N‐terminal kinase
KAISO: Kaiso (BTB and CNC homology 1)
LIN52: protein lin‐52 homolog
MAP1B: microtubule‐associated protein 1B
MAPK: mitogen‐activated protein kinase
MEF2D: myocyte enhancer factor 2D
mnb: minibrain
MRD7: mental retardation, autosomal dominant 7
NAPA: N‐terminal autophosphorylation accessory domain
NFAT: nuclear factor of activated T cells
NFT: neurofibrillary tangles
NLS: nuclear localization signal
NMDA: N‐methyl‐D‐aspartate
NRSF: neuron‐restrictive silencer factor
OLIG2: oligodendrocyte transcription factor 2
p300: E1A binding protein p300
p53: tumor protein 53
PD: Parkinson's disease
PEST: proline, glutamic acid, serine, threonine‐rich domain
PI3K: phosphoinositide 3‐kinase
PROTAC: proteolysis‐targeting chimera
RCAN1: regulator of calcineurin 1
REST: RE1‐silencing transcription factor
RNAPII: RNA polymerase II
RNF169: ring finger protein 169
ROS: reactive oxygen species
SAR: structure–activity relationship
SF3b1: splicing factor 3B, Subunit 1
SIRT1: sirtuin 1
SPRED1: sprouty‐related EVH1 domain‐containing protein 1
SPRED2: Sprouty‐related EVH1 domain‐containing protein 2
SPRY2: Sprouty 2
SRSF6: serine/arginine‐rich splicing factor 6
STAT3: signal transducer and activator of transcription 3
T1D: type 1 diabetes
T2D: type 2 diabetes
TRAF2: TNF receptor‐associated factor 2
TSC1/TSC2: tuberous sclerosis complex

## 1 Introduction

The dual‐specificity tyrosine‐phosphorylation‐regulated kinase (DYRK) family represents an evolutionarily conserved group of protein kinases that play pivotal roles across a spectrum of cellular processes from yeast to humans (Arslanhan et al. 2025). Among the DYRK family, DYRK1A is the most intensively investigated member due to its gene residing within the Down syndrome critical region (DSCR) on human chromosome 21 (Pathak et al. 2018). DYRK1A is dosage‐sensitive with small changes in its expression or activity, producing marked clinical effects. DYRK1A overexpression in Down syndrome (DS, trisomy 21) causes intellectual disability, neurodegeneration and congenital heart defects, while haploinsufficiency (MRD7) produces developmental delay, intellectual disability, microcephaly and seizures (Tran et al. 2020; Courcet et al. 2012; Brault et al. 2021; Bronicki et al. 2015).

The discovery of DYRK kinase emerged from cross‐species studies that linked *Drosophila* genetics to vertebrate biochemistry. The identification of the “*Drosophila minibrain*” (*mnb*) gene in 1995 revealed that this protein kinase is crucial for postembryonic neurogenesis (Tejedor et al. 1995). Subsequently, cloning of broadly expressed DYRK cDNA from rat brain in 1996 revealed a conserved catalytic domain that, despite being only distantly related to other mammalian protein kinases, shares about 85% amino acid sequence identity with *Drosophila mnb* within the kinase domain (Dierssen and de Lagrán 2006).

By the late 1990s, homologs in humans were identified, revealing a mammalian DYRK family composed of five members: DYRK1A, DYRK1B, DYRK2, DYRK3 and DYRK4 (Yoshida and Yoshida 2023). The term “DYRK” reflects their “dual‐specificity” behaviour; these kinases autophosphorylate on tyrosine residues while primarily phosphorylating serine and threonine residues on substrates (Nguyen et al. 2017). A conserved activation‐loop tyrosine (e.g., Tyr321 in DYRK1A) is autophosphorylated during protein synthesis and folding, producing a constitutively active enzyme that does not require upstream kinases for activation (Soundararajan et al. 2013).

The centrality of DYRK1A in numerous human diseases has driven intensive efforts to delineate its biological roles and develop selective therapeutics with a primary goal of producing highly specific DYRK1A inhibitors. Achieving selectivity is a significant challenge as the ATP‐binding pocket is structurally conserved across many kinases, complicating discrimination between DYRK1A and off‐target enzymes. Nonetheless, several candidate inhibitors with favorable selectivity profiles have emerged and some have advanced into early clinical evaluation (Boni et al. 2020).

Despite these advances, several knowledge gaps remain. To address these, a detailed elucidation of the structural determinant of DYRK1A activation and substrate recognition is required. Additionally, SAR insights are essential for refining inhibitor scaffolds, and robust pharmacodynamic biomarkers that reliably confirm in vivo target engagement are equally required. Therefore, this review aims to leverage current evidence on regulatory roles and various molecular pathways of DYRK1A to inform rational inhibitor design and translational validation. This includes exploring future prospects such as the development of specific biomarkers, novel therapies and next‐generation kinase inhibitors with enhanced selectivity and efficacy.

## 2 Classification of DYRK Kinases

DYRKs belong to the CMGC group of eukaryotic protein kinases, which also contains Cyclin‐dependent kinases (CDKs), Mitogen‐activation protein kinases (MAPKs), Glycogen synthase kinases (GSKs) and CDK‐like kinases (CLKs) (Boni et al. 2020). Within the DYRK family, members segregate into two subgroups based on sequence elements located outside the conserved catalytic core:

*Class I DYRKs*, comprising of DYRK1A and DYRK1B, are distinguished by multiple regulatory domains that facilitate their translocation to the nucleus and regulate their transcriptional activity. These kinases possess nuclear localization signals (NLS) (Bhansali et al. 2021) directing their import into the nucleus, executing critical roles in transcriptional control. Complementing this nuclear targeting function, i.e., the PEST‐rich region‐ proline, glutamic acid, serine, threonine‐rich domain that acts as a degron, enabling rapid proteasomal degradation pathways (Ananthapadmanabhan et al. 2023). This regulatory architecture allows for precise temporal control of DYRK1A and DYRK1B levels during critical embryonic and early postnatal developmental windows. Although DYRK1A and DYRK1B share approximately 85% identity within their catalytic kinase domains, their C‐terminal tails diverge critically. DYRK1A uniquely contains a histidine‐rich repeat that directs it to nuclear speckles for splicing regulation in neurogenesis, while DYRK1B lacks this repeat and instead mediates myoblast quiescence and peripheral metabolic functions, including adipogenesis (Rammohan et al. 2022).

*Class II DYRKs*, includes DYRK2, DYRK3, and DYRK4, differ fundamentally from Class I members in their subcellular localization and regulatory architecture. These kinases lack the NLS and PEST domains characteristic of Class I members, resulting in their primary localization to the cytoplasm (Aranda et al. 2011). Notably, DYRK2 possesses a nuclear import capability and can shuttle between nuclear and cytoplasmic compartments under specific cellular conditions (Singh and Lauth 2017). Class II members are distinguished by a conserved N‐terminal autophosphorylation accessory (NAPA) domain, comprising two structurally and functionally distinct subdomains: NAPA1 and NAPA2 (Papadopoulos et al. 2011).

All DYRKs share a conserved catalytic kinase domain and an upstream DYRK Homology (DH) box motif that governs subcellular distribution, substrate selection, and functional diversity (Detro‐Dassen et al. 2025).

## 3 Molecular and Structural Biology of DYRK1A

### 3.1 Gene and Genomic Organization

The human *DYRK1A* gene maps to chromosome 21 (HSA21) at 21q22.2 or 21q22.13 within the “DSCR”. It spans approximately 151 kilobases and is composed of 15 exons. Alternative splicing generates multiple protein isoforms (754–780 amino acid), all retain kinase activity, but only the brain predominant 763‐amino acid variant preserves the C‐terminal histidine‐rich nuclear speckles‐targeting signal essential for splicing factor regulation during neurogenesis. Since, DYRK1A resides within DSCR, altered gene dosage is considered a major contributor to phenotypes observed in DS (Laham et al. 2021).

DYRK1A displays tightly regulated spatio‐temporal expression, peaking around birth, and declining to lower levels in adulthood. Expression is especially high in the cerebellum, olfactory bulb, and hippocampus (Arbones et al. 2019; Martí et al. 2003). It is highly conserved (> 99% identity with rodent orthologues); its closest paralog, DYRK1B, shares 73.9% sequence identity (85% within the kinase domain) (Hämmerle et al. 2008).

#### 3.1.1 Regulatory Control of DYRK1A

DYRK1A is controlled by integrated transcriptional, post‐transcriptional, and protein‐level mechanisms that produce precise developmental and tissue‐specific expression. At the transcriptional level, REST/NRSF binds a neuron‐restrictive silencer element (NRSE) in the *DYRK1A* promoter region (specifically from −833 to −815 bp) to modulate transcription (Negrini et al. 2013). This forms a negative feedback loop in which DYRK1A dosage can influence REST stability and transcriptional activity via ubiquitin‐mediated degradation (Lu et al. 2011).

The truncated p53 isoforms, p44, promote DYRK1A transcription, while full‐length p53 induces miR‐1246 to suppress *Dyrk1a* mRNA. miR‐199b also targets DYRK1A in cardiac hypertrophy (Pehar et al. 2014).

E2F1 and chromosome‐21 encoded OLIG2 further shape promoter activity (Liu et al. 2015), while SPRED1/2 modulate catalytic output (Becker and Sippl 2011), and MEF2D couples transcriptional regulation to substrate function (Liu, Nelson, et al. 2014).

Genome‐wide analyses show DYRK1A preferentially occupies promoters of actively transcribed genes (TCTCGCGAGA motif), acting with KAISO/ZBTB33, CHD2, and BRCA1 to drive transcription (Vona et al. 2015).

### 3.2 DYRK1A: Enzyme Architecture and Functional Domains

DYRK1A kinase contains several distinct regions that coordinate localization, catalytic activity, regulation and turnover:

*N‐terminal region*: Contains a bipartite nuclear localization signal (NLS1) and a conserved DYRK homology (DH) box that serves three functions: scaffolding irreversible co‐translational Tyr321 autophosphorylation, stabilizing the kinase domain fold, and facilitating substrate recognition specificity (Widowati et al. 2018).

*Catalytic kinase domain*: This central domain is responsible for DYRK1A's kinase activity. The N‐terminal lobe present in the kinase domain is composed of five antiparallel β‐strands and a regulatory αC‐helix, and a larger C‐terminal lobe of multiple α‐helices linked by a hinge region that forms the ATP‐binding pocket. A MAP kinase insertion within the C‐lobe differentiates DYRK1A from other DYRK family members and contributes to its unique catalytic properties (Kentrup et al. 1996).

**FIGURE 1:**
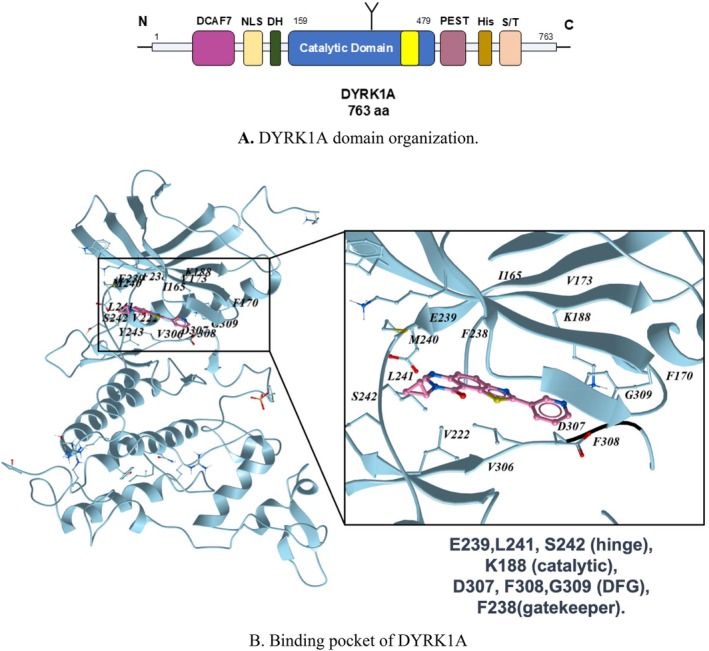
(A) The first image demonstrates a schematic DYRK1A domain map showing N‐terminal DCAF7, NLS, DH, catalytic, PEST, His and S/T regions. The ‘Y’ symbol on the catalytic domain denotes the auto‐phosphorylated activation‐loop residue Tyr321 and (B) The second image illustrates the ATP‐binding pocket of DYRK1A and its associated amino acid residues responsible for interaction. The structure was rendered from PDB (6QU2) and the pink molecule represents the 8‐cyclopropyl‐2‐pyridin‐3‐yl‐[1,3]thiazolo[5,4‐f]quinazolin‐9‐one the actual co‐crystallized ligand. Created using MolSoft ICM Pro.

*C‐terminus*: Features a PEST sequence (rich in proline, glutamic acid, serine, and threonine) that targets the protein for proteasomal degradation, regulating its intracellular half‐life. The pathogenic truncations in haploinsufficiency syndrome that eliminate this motif disrupt kinase dosage without restoring nuclear function (Jarhad et al. 2018). A histidine‐rich repeat (His) encoding a nuclear speckle targeting signal (STS) that cooperates with the NLS to direct DYRK1A specifically to nuclear speckles, enabling phosphorylation of splicing factors such as SRSF6 and SRp55 (Figure 1A) (Huang et al. 2023).

#### 3.2.1 ATP‐Binding Site of DYRK1A and Its Key Interacting Residues

The ATP‐binding site of DYRK1A lies between the N‐ and C‐terminus lobes of the kinase domain and constitutes the structural and functional core that drives catalysis and serves as the primary target for small‐molecule inhibitors. The hinge region that connects the two lobes forms a boundary of the pocket and presents peptide‐backbone, hydrogen‐bond donors and acceptors that many inhibitors emulate to gain binding affinity and specificity. The key structural determinants of this pocket include Lys188, a catalytic residue essential for phosphoryl transfer, and Phe238, the gatekeeper residue controlling access to a contiguous hydrophobic back pocket. Adjacent residues Glu239 (gk + 1) and Leu241 (gk + 3) contribute to important side‐chain interactions with inhibitors that occupy the hinge region. Other residues such as Phe170, Ser242, Asn292, and Asp307 collectively define the electrostatic and hydrophobic character of the pocket, shaping ligand complementarity. The DFG (aspartate‐phenylalanine‐glycine) motif and its surrounding pocket create the activation loop microenvironment, a conformationally sensitive element that influences kinase activity and inhibitor binding (Figure 1B) (Shahroz et al. 2022). The DFG motif within the activation loop exists in two principal conformations that fundamentally determine inhibitor binding mode. In the DFG‐in (active) conformation, the Asp residue orients inward to coordinate two Mg^2+^ ions essential for ATP‐dependent phosphotransfer, and the kinase is catalytically competent. In contrast, the DFG‐out (inactive) conformation involves Asp and Phe swapping positions: Asp moves approximately 5 Å away from the ATP‐binding site while Phe rotates inward, rendering the kinase catalytically incompetent. Critically, this rearrangement expands the binding cavity and exposes an adjacent hydrophobic allosteric pocket absent in the DFG‐in state. Type‐I (ATP‐competitive) inhibitors bind exclusively within the DFG‐in ATP pocket via hinge‐region hydrogen bonds, whereas Type II inhibitors exploit the DFG‐out allosteric pocket, generally achieving improved selectivity through engagement of a region with lower sequence conservation across kinases (Umezawa and Kii 2021).

### 3.3 Post‐Translational Modifications of DYRK1A

#### 3.3.1 Phosphorylation of DYRK1A

Phosphorylation is a crucial mechanism regulating DYRK1A activity, intracellular distribution, and interactions. DYRK1A is a dual‐specificity kinase; it autophosphorylates for self‐activation, yet it phosphorylates its substrates on serine and threonine residues (Walte et al. 2013).

##### 3.3.1.1 Autophosphorylation for Activation

**FIGURE 2:**
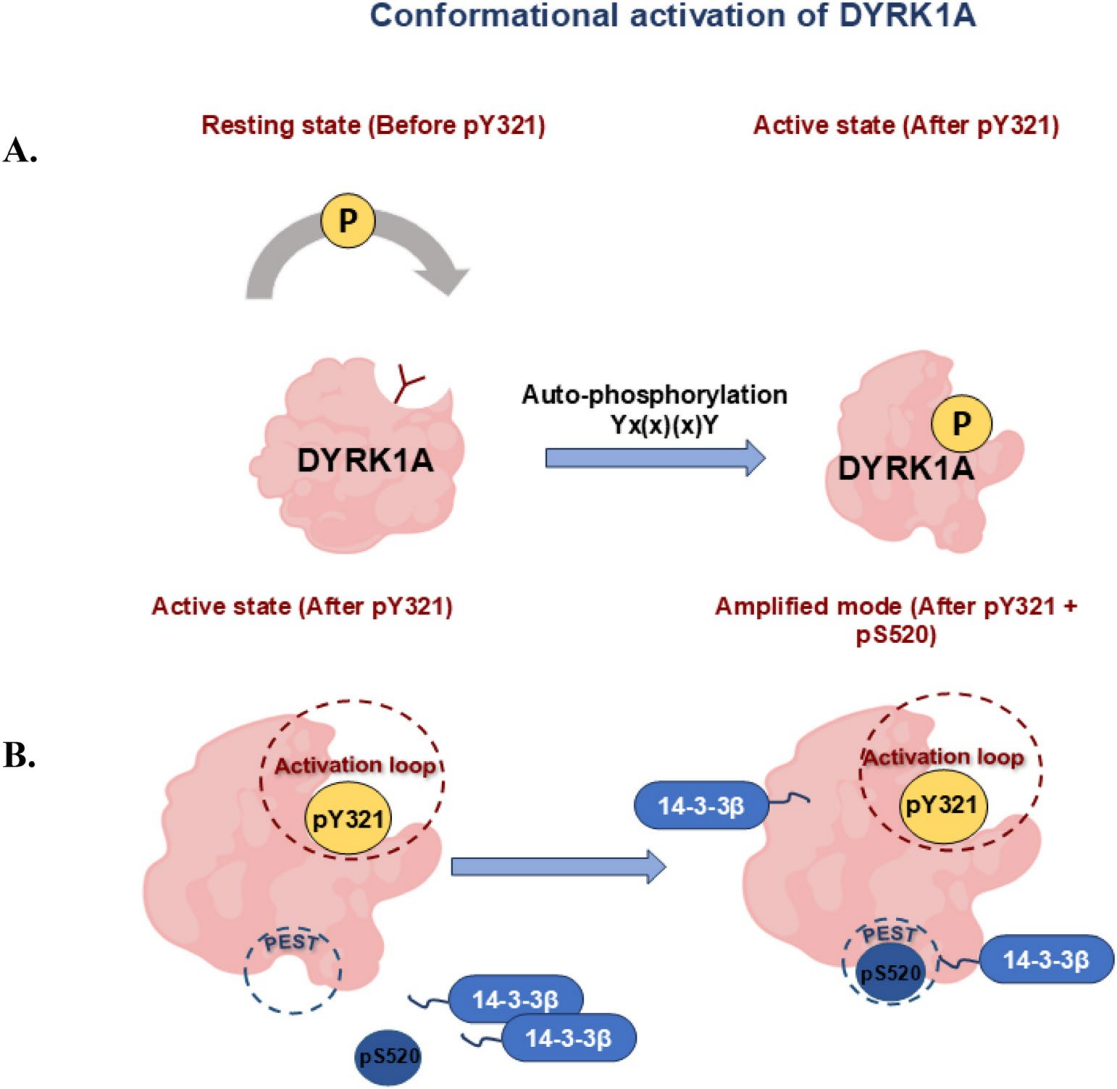
(A) The image represents DYRK1A activation from resting (before pY321) to active (after pY321) via auto‐phosphorylation at Yx(x)(x)Y (where ‘Y’ denotes Tyr residue and ‘x’ is any amino acid) with transfer of a phosphate group. (B) DYRK1A shifts from active (pY321) to amplified mode when phosphor‐Ser520 (pS520, auto‐phosphorylated S520 of DYRK1A) recruits 14‐3‐3β proteins, stabilizing enhanced signaling and sustained activation.

*Tyrosine 321* (*Y321*) Autophosphorylation at tyrosine 321 (isoform 1) within the activation loop is an irreversible co‐translational event: the nascent DYRK1A polypeptide, while still being translated on the ribosome, phosphorylates its own activation‐loop tyrosine via a transient intermediate requiring the DH box as a structural scaffold. Once translation terminates, this tyrosine kinase activity is irreversibly abolished and the fully‐folded mature enzyme functions exclusively as a serine–threonine kinase (Figure 2A) (Lochhead et al. 2005).

*Serine 520* (*S520*) Autophosphorylation of serine residue S520 within the PEST domain also occurs through an intramolecular mechanism. The phosphorylation at this site is required for the recruitment of the scaffold protein 14‐3‐3β, and binding of 14‐3‐3β enhances DYRK1A catalytic activity by as much as 100% (Kim et al. 2004). This 14‐3‐3β‐dependent catalytic activity enhancement is mechanistically distinct from Y321 autophosphorylation and does not require prior Y321 phosphorylation. Two 14‐3‐3 binding sites exist on DYRK1A, with Ser‐520 being one and a second located within the N‐terminal 150 amino acids (Figure 2B) (Alvarez et al. 2007).

*Compartment‐Specific Phosphorylation*: DYRK1A displays location‐dependent phosphorylation profiles.

*Cytosolic DYRK1A* bears single phosphorylation (Y321; pI 8.7), with λ‐phosphatase removal shifting pI to 9.0.

*Cytoskeletal DYRK1A*: This form of DYRK1A is more acidic, with observed pI values between 7.2 and 8.2 and is phosphorylated at multiple sites. Mass spectrometric analysis of mouse DYRK1A identified Ser47 and Ser49 as cytoskeletal‐compartment‐specific phosphorylation sites distinct from the activation‐loop residue Tyr321; however, their precise functional roles remain to be experimentally established (Himpel et al. 2001).

**FIGURE 3:**
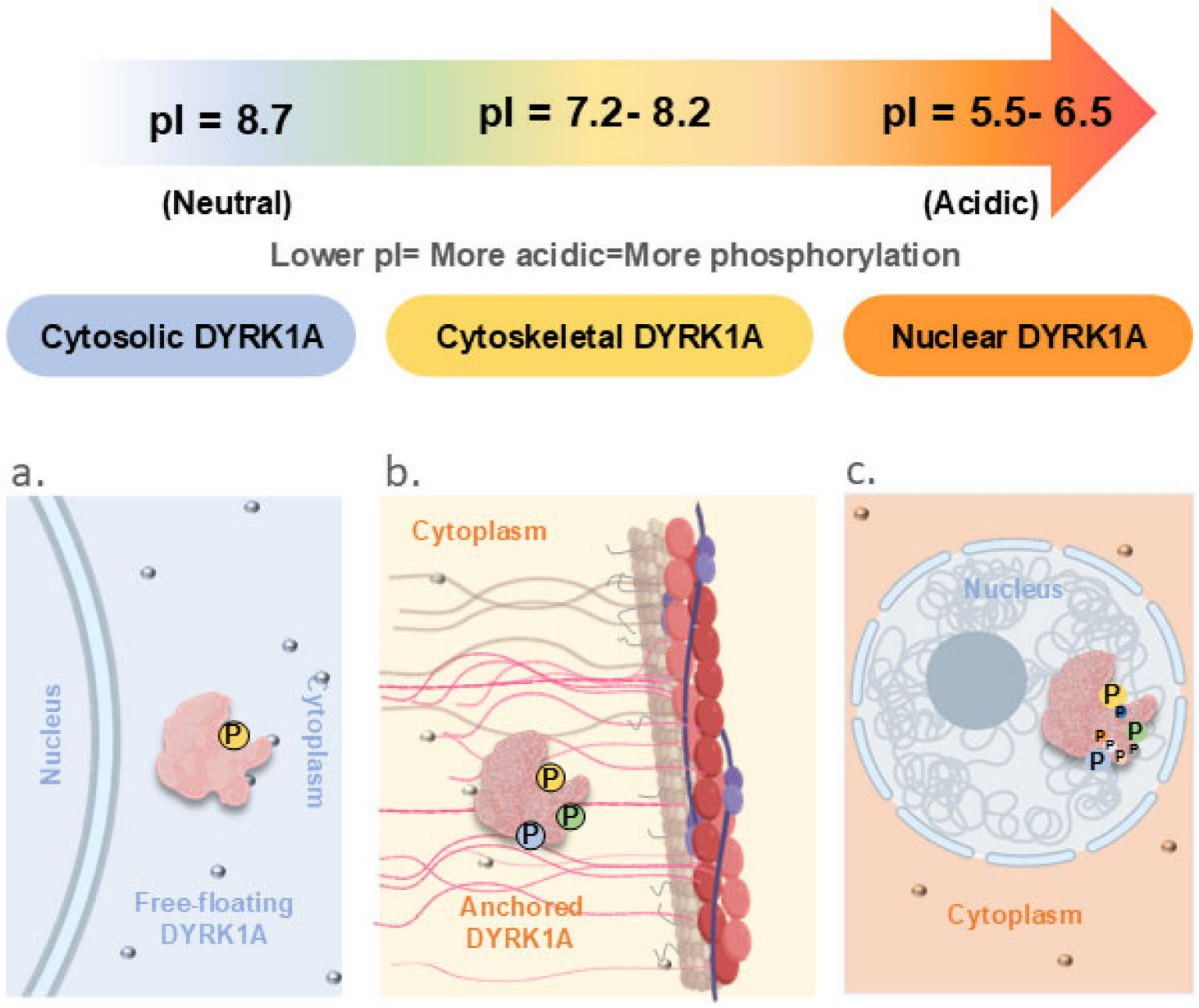
Gradient shows DYRK1A pI decreasing from 8.7 to 5.5–6.5, linking cytosolic, cytoskeletal, nuclear localization with progressively increased phosphorylation.

*Nuclear DYRK1A* shows a pronounced acidic shift, isoelectric points around 5.5–6.5 and exists as several isoforms consistent with differential phosphorylation states (Figure 3) (Kaczmarski et al. 2014).

#### 3.3.2 Ubiquitination of DYRK1A

DYRK1A is subjected to K63‐linked ubiquitination catalyzed by E3 ligase TRAF2. TRAF2 recognizes a PVQE motif located between the PEST sequence and histidine repeat of DYRK1A (Zhang et al. 2021). This K63‐linked modification drives partial relocalization of DYRK1A to vesicle membranes, notably to detergent‐insoluble vesicular compartment, a step required for its vesicle‐related functions and for phosphorylation of substrates involved in vesicle dynamics such as Sprouty 2 (SPRY2), synaptojanin 1, endophilin 1, and dynamin 1 (Murakami et al. 2009; Murakami et al. 2012).

## 4 Biological Roles of DYRK1A

### 4.1 Role in Cell Cycle Regulation

DYRK1A enforces cell cycle arrest through a coordinated molecular brake operating simultaneously at three levels: degrading pro‐proliferative signals (Cyclin D1), stabilizing anti‐proliferative signals (p27Kip1), and establishing transcriptional quiescence (DREAM complex via LIN52). These are not independent phosphorylation events but components of a single reinforcing program in which each action strengthens the others (Soppa et al. 2014).

Mechanistically, DYRK1A phosphorylates Cyclin D1 at Thr286, targeting it for proteasomal degradation, thereby lowering Cyclin D1 abundance. Concurrently, phosphorylation of p27Kip1 at Ser10 stabilizes this endogenous CDK2/CDK4 inhibitor and increases its cellular levels (Najas et al. 2015).

DYRK1A drives formation of the DREAM complex by phosphorylating LIN52 on Ser28. The DREAM transcriptional repressor then suppresses cell‐cycle gene expression to induce quiescence. DYRK1A‐dependent DREAM assembly has been implicated in tumor cell dormancy in ovarian cancer and in quiescence of gastrointestinal stromal tumor cells (Litovchick et al. 2011).

DYRK1A phosphorylates p53 (Ser15), upregulating p21CIP1 and limiting G1‐S progression (Hämmerle et al. 2011); however, it can indirectly suppress p53 via Sirtuin 1 (SIRT1)‐dependent deacetylation (Guo et al. 2010). A regulatory feedback loop closes when p53 induces miR‐1246 to suppress DYRK1A (Zhang et al. 2011).

### 4.2 Role in Neurodevelopment

DYRK1A is transiently expressed in neuronal progenitors, promoting cell‐cycle exit; overexpression accelerates premature differentiation (Yabut et al. 2010). DYRK1A supports neuronal integrity through two sequential programs. The first builds the dendritic scaffold by phosphorylating cytoskeletal proteins TAU (Thr212), MAP1B, and α‐tubulin to regulate morphogenesis. The second maintains functional activity by phosphorylating endocytic proteins Dynamin1, Amphiphysin1, and Synaptojanin1 to sustain synaptic vesicle recycling (Murakami et al. 2009; Huang et al. 2004; Murakami et al. 2006). Structural assembly and functional maintenance are therefore not parallel but coupled, reinforcing each other (Wang et al. 2023). In *Drosophila*, the DYRK1A ortholog minibrain performs analogous roles in promoting efficient synaptic vesicle turnover (Lowe et al. 2019; Cisternas et al. 2025).

DYRK1A is also necessary for ciliogenesis; its depletion in *Xenopus* embryos causes reduced forebrain size and abnormal cell‐cycle progression during brain formation, consistent with impaired microtubule dynamics (Willsey et al. 2020).

### 4.3 Role in Transcriptional Regulation

**FIGURE 4:**
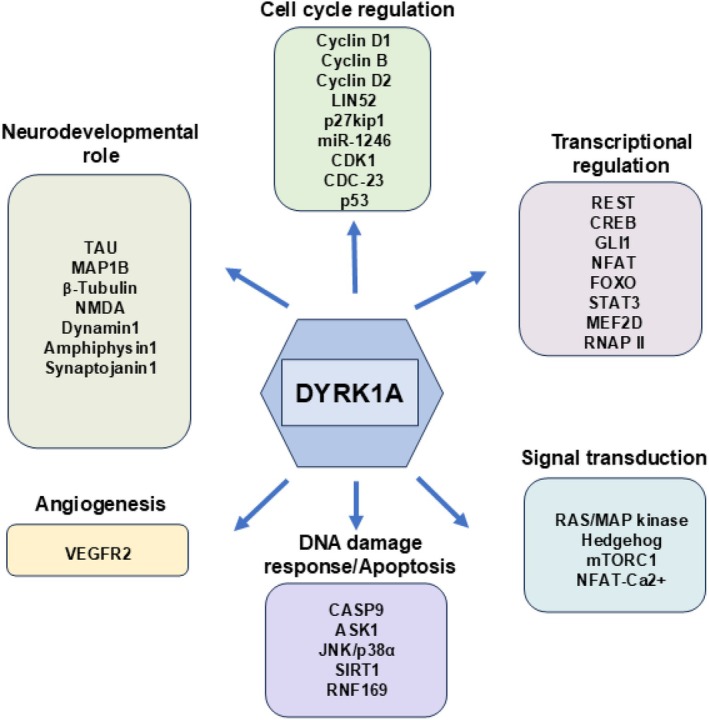
DYRK1A centrally regulates neurodevelopment, angiogenesis, cell cycle, DNA damage response, transcription and signaling, thereby connecting diverse substrates and pathways in cells.

DYRK1A controls gene expression through kinase‐dependent and kinase‐independent mechanisms, acting as a selective RNA polymerase II (RNAPII) CTD kinase (phosphorylating Ser2 and Ser5) to regulate ribosome biogenesis, transcriptional control, and myogenic programs (Figure 4) (Vona et al. 2015).

A broad set of DYRK1A‐transcription factor interactions further diversified by its transcriptional evidences are summarized in Table 1.

**TABLE 1:** DYRK1A interaction with various transcription factors.

S. no.	Transcription factor	Interaction and effect with DYRK1A	References
1.	REST	It binds the DYRK1A promoter to drive its transcription and elevated DYRK1A reciprocally decreases REST protein stability and its transcriptional activity, forming a negative feedback loop	Lu et al. (2011)
2.	CREB	DYRK1A enhances CREB activity, thereby increasing CREB‐dependent transcription	Choi and Chung (2011)
3.	GLI1	DYRK1A enhances GLI1 transcriptional activity by direct phosphorylating the GLI1 protein	Mao et al. (2002)
4.	NFAT	DYRK1A phosphorylates NFAT family members to drive cytoplasmic retention and transcriptional silencing in most cellular contexts. However, in VEGF‐stimulated endothelial cells and GBM, it paradoxically amplifies NFAT signaling, illustrating that NFAT regulation is context‐dependent and co‐regulator determined, not constitutively suppressive	Rozen et al. (2018)
5.	FOXO	DYRK1A phosphorylates FOXO1 (FKHR), modulating its role in cell cycle control and cellular proliferation	Bhansali et al. (2021)
6.	STAT3	DYRK1A phosphorylates STAT3, modulating STAT3‐driven signaling pathway	
7.	MEF2D	Enhances DYRK1A transcription via promoter activation, preferentially increasing expression of isoform 5 and thereby elevating DYRK1A kinase activity	Wang et al. (2017)

### 4.4 Role in Signal Transduction Pathways

DYRK1A participates in numerous signal transduction pathways, modulating cellular responses to diverse external and internal cues (see Table 2).

**TABLE 2:** DYRK1A involvement in various signaling pathways and mechanism of modulation.

S. no	Signaling pathway	Transduction mechanism by DYRK1A	References
1.	RAS/MAP kinase pathway	Facilitates NGF‐driven differentiation of PC12 cells by enhancing the Ras/MAPK cascade independently of its kinase activity, extending ERK activation through assembly of a multiprotein complex with Ras, B‐Raf and MEK1	Kelly and Rahmani (2005)
2.	NFAT signaling	Primarily acts to restrain NFAT nuclear accumulation, yet in VEGF‐stimulated primary endothelial cells it enhances NFAT signaling and thereby supports angiogenic responses	Gwack et al. (2006)
3.	Hedgehog (Hh) Signaling	Enhances GLI1 transcriptional activity by direct phosphorylation while concurrently suppressing Hedgehog signaling indirectly through modulation of Actin cytoskeleton regulators	Schneider et al. (2015)
4.	mTORC1 pathway	DYRK1A increases cell size and activates mTORC1 by binding the TSC1/TSC2 complex and phosphorylating TSC2 at Thr1462, which inhibits TSC function and thereby relieves suppression of mTORC1	Wang et al. (2024)
5.	DNA Damage response (DDR) and Apoptosis	DYRK1A modulates multiple cell death and stress‐response pathways. It phosphorylates SIRT1 at Thr533 to enhance SIRT1‐dependent p53 deacetylation, phosphorylates RNF169 to influence 53BP1 displacement and double‐strand break repair and limits intrinsic apoptosis by phosphorylating caspase‐9 at Thr125. Conversely, under cellular stress DYRK1A can activate ASK1, triggering a JNK/p38α cascade that promotes apoptosis and when overexpressed, it may itself induce cell death	Guo et al. (2010); Menon et al. (2019)
6.	Angiogenesis	DYRK1A promotes angiogenesis in endothelial cells by modulating Ca^2+^/NFAT signaling and stabilizing VEGFR2	Rozen et al. (2018)

## 5 Pathological Implications of DYRK1A in Various Diseases

DYRK1A pathology arises through three mechanistically distinct modes of dysregulation. The first is aberrant temporal expression: either premature overexpression during neurogenesis (DS) or insufficient expression during critical developmental windows (MRCD7) (Murphy et al. 2024), both of which disrupt the narrow kinase dosage required for normal neural progenitor timing. The second is chronic up‐regulation in post‐developmental adult tissue, as observed in Alzheimer's disease (AD) (Shukla et al. 2023), Parkinson's disease (PD) (Yong et al. 2023) and most solid tumors, where sustained hyperphosphorylation of substrates that would be transiently modified under normal expression produces irreversible cellular damage. The third is alternatively expressed isoforms: the brain‐predominant 763‐amino acid variant retains the C‐terminal histidine‐rich speckle‐targeting signal essential for splicing factor regulation, while shorter isoforms lacking this domain redirect kinase activity toward cytoplasmic substrates, contributing to tau exon 10 imbalance in AD and aberrant TNNT2 splicing in DS cardiac tissue. Each sub‐section below maps its disease to one or more of these three categories (Quiñones‐Lombraña and Blanco 2019).

### 5.1 Pathological Alterations in Neurodevelopmental and Haploinsufficiency Syndromes

Both DYRK1A overexpression (DS, trisomy 21) and haploinsufficiency (MRD7) disrupt neurogenesis through the same cell cycle regulators p53/p21 and Cyclin D1, but in opposing directions, reflecting a narrow functional activity window outside which neurodevelopmental dysrhythmia results (Soppa et al. 2014).

#### 5.1.1 DS: The Cascade of Kinase Overexpression

In DS, DYRK1A overexpression of approximately 1.5‐fold operates through three non‐overlapping pathological mechanisms‐ cell cycle dysregulation, NFAT suppression and splicing disruption, each of which is described below (Deboever et al. 2022).

##### 5.1.1.1 Aberrant Regulation of Neuronal Cell Cycle and Differentiation

Elevated DYRK1A disrupts this equilibrium by promoting premature cell cycle exit and accelerated differentiation (Yabut et al. 2010). The molecular mechanism involves DYRK1A phosphorylating Cyclin D1, marking it for degradation and preventing cells from progressing through the G1/S checkpoint (Murphy et al. 2024). Additionally, DYRK1A activates the p53/p21 pathway through phosphorylation of p53 at Ser15, triggering cell‐cycle arrest in embryonic neurons. These combined effects reduce the total number of neurons generated, particularly affecting brain regions critical for cognition such as the cortex and hippocampus (Zhang et al. 2011).

##### 5.1.1.2 NFAT Signaling Dysregulation and Collaborative Pathogenesis

**FIGURE 5:**
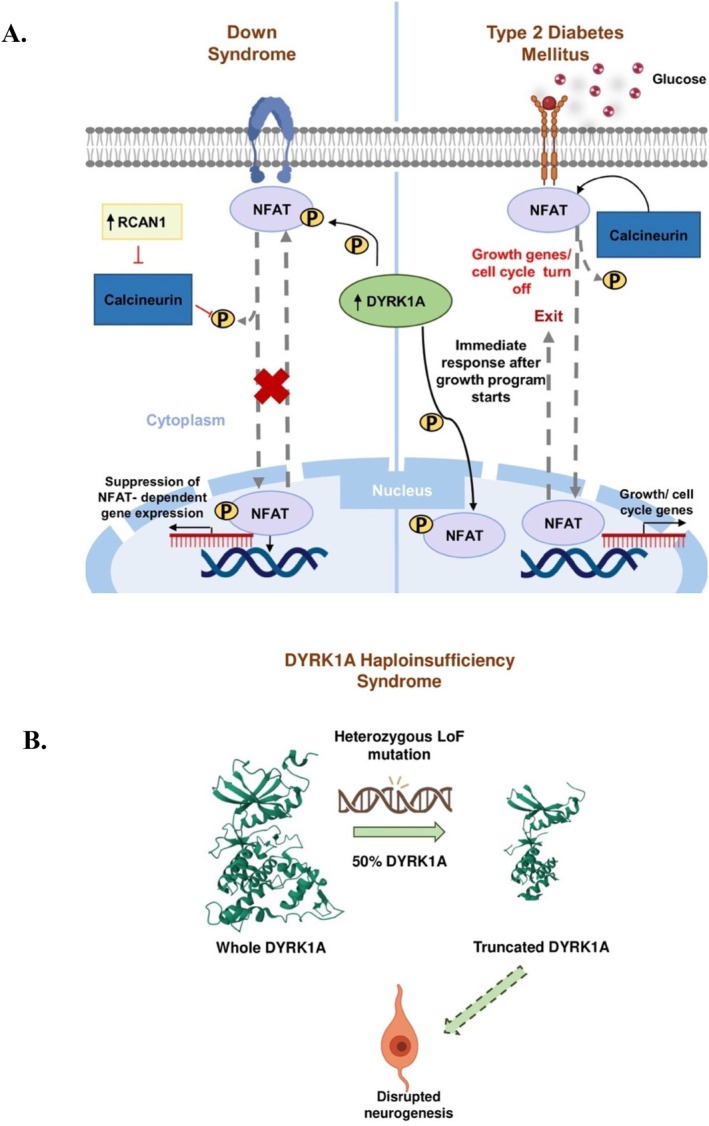
(A) Integrative model of DYRK1A‐dependent NFAT signaling across two disease contexts. Left panel (Down syndrome): DYRK1A and RCAN1 hyperactivity, phosphorylates NFAT blocking nuclear entry and thereby suppressing NFAT‐dependent gene expression. Right panel (Type 2 diabetes mellitus): High glucose activates membrane signaling, DYRK1A phosphorylates NFAT, causing nuclear exit, turning off growth and cell‐cycle genes, thereby repressing transcription. (B) Heterozygous DYRK1A loss produces truncated protein, 50% expression, causing disrupted neurogenesis in developing brain neurons.

DYRK1A acts as a potent repressor of NFAT (Nuclear factor of activated T cells) transcription factors, proteins that normally promote the transcription of genes essential for neuronal survival and function. In DS, this repression is amplified by concurrent overexpression of RCAN1 (Regulator of Calcineurin 1), which also inhibits NFAT activation. This synergistic suppression of NFAT signaling substantially impairs normal brain development and contributes to intellectual disability. The same DYRK1A‐calcineurin‐NFAT suppression axis operates in the pancreatic β‐cell context of Type 2 diabetes mellitus, where chronic DYRK1A hyperactivity locks β‐cells in a quiescent transcriptional state via nuclear NFAT exit. Both contexts are illustrated in Figure 5A (Dirice et al. 2016; Duchon and Herault 2016).

##### 5.1.1.3 Cardiovascular Manifestations

A clinical hallmark of DS is the high incidence of congenital heart defects (CHD), affecting approximately 40%–50% of individuals (Benhaourech et al. 2016). The pathological mechanism involves DYRK1A disrupting alternative RNA splicing in cardiac tissue. Specifically, DYRK1A overexpression in cardiomyocytes perturbs the normal splicing of the TNNT2 gene, which encodes cardiac troponin T (cTnT), a critical contractile protein. DYRK1A accomplishes this by modulating the activity of SRSF6, a splicing regulator protein (Quiñones‐Lombraña and Blanco 2019). The resulting aberrant cTnT isoforms impair cardiac muscle contraction and render these cells hypersensitive to cardiotoxic drugs, such as anthracycline chemotherapy agents commonly used in cancer treatment (Lu and Yin 2016).

#### 5.1.2 DYRK1A Haploinsufficiency Syndrome (MRD7, IDD, ASD): Consequences of Reduced Kinase Activity

While DS results from DYRK1A excess, heterozygous DYRK1A loss‐of‐function (LoF) mutations (designated as DYRK1A haploinsufficiency syndrome [DHS], also designated MRD7 [Mental retardation autosomal dominant 7]) produce truncated or catalytically null proteins, causing intellectual disability, microcephaly, seizures, and motor incoordination, underscoring strict dosage requirements. These clinical features underscore the absolute requirement for appropriately dosed DYRK1A in normal brain development (Figure 5B) (Brault et al. 2021; Arranz et al. 2019; Ji et al. 2015).

##### 5.1.2.1 Molecular Basis of Microcephaly and Circuit Defects

The reduced DYRK1A activity impairs the precise temporal coordination of neural progenitor cell proliferation and differentiation. This neurodevelopmental dysrhythmia (the disruption of normal developmental timing) compromises the generation of sufficient neuronal numbers and impairs the assembly of properly organized cortical circuits. Critically, DYRK1A LoF impairs the growth of cortical pyramidal neurons by disrupting growth factor signaling pathways essential for neuronal maturation and axonal extension (Shih et al. 2023; Levy et al. 2021).

##### 5.1.2.2 Mitochondrial Link in DYRK1A Syndrome

Recent evidence reveals a mechanistic link between DYRK1A and mitochondrial function. DYRK1A phosphorylates and regulates TOM70, a translocase protein located in the outer mitochondrial membrane responsible for importing proteins into mitochondria. Loss of DYRK1A function disrupts this mitochondrial protein import system, leading to the accumulation of unimported proteins in the cytoplasm and impaired mitochondrial protein synthesis. This mitochondrial dysfunction may explain why DYRK1A haploinsufficiency manifests with clinical features overlapping mitochondrial cytopathies like motor impairments, developmental delay, autism, and intellectual disability (Marada et al. 2024).

### 5.2 DYRK1A in Neurodegenerative Disorders

In contrast to developmental diseases driven by aberrant temporal expression, the neurodegenerative conditions reflect age‐dependent DYRK1A up‐regulation in adult tissue, where sustained hyperphosphorylation of aggregation‐prone substrates drives cumulative protein misfolding and neuronal dysfunction.

#### 5.2.1 AD: The Bridge Between Amyloid and Tau

AD is pathologically characterized by extracellular deposits of β‐amyloid (Aβ) peptide and intracellular neurofibrillary tangles (NFTs) composed of hyperphosphorylated tau protein. Elevated DYRK1A activity serves as a molecular bridge linking these two pathological processes (Wegiel et al. 2011).

##### 5.2.1.1 Pathological Pathway I: Amyloid Precursor Protein Processing

DYRK1A phosphorylates amyloid precursor protein (APP) at threonine 668, a modification that facilitates APP cleavage by β‐secretase (BACE1) and γ‐secretase proteases. These cleavage events generate neurotoxic Aβ‐40 and Aβ‐42 peptides that accumulate in extracellular plaques (Zhang et al. 2020). This creates a pathological feedforward loop: as Aβ accumulates, it triggers upregulation of DYRK1A expression, which further amplifies Aβ production (Branca et al. 2017). Compounding this problem, DYRK1A phosphorylates and downregulates neprilysin (NEP), the primary protease responsible for degrading and clearing Aβ peptides, thereby prolonging amyloid accumulation and toxicity (Coutadeur et al. 2015).

##### 5.2.1.2 Pathological Pathway II: Tau Hyperphosphorylation and Splicing Aberrations

DYRK1A phosphorylates tau protein at threonine 212, acting as a priming kinase; that is, it creates a recognition site for glycogen synthase kinase 3β (GSK3β), which then catalyzes additional tau phosphorylation events. These hyperphosphorylated tau molecules progressively aggregate into the insoluble NFTs characteristic of AD. Beyond direct phosphorylation, DYRK1A regulates the relative abundance of tau protein isoforms by controlling alternative splicing through interaction with the splicing factor SRp55. Specifically, elevated DYRK1A promotes inclusion of tau exon 10, shifting the balance from 3R tau (3‐repeat tau) toward 4R tau (4‐repeat tau) isoforms, an imbalance that accelerates tau aggregation and is seen in other tauopathies. This splicing dysregulation exemplifies the third mode of DYRK1A pathology, alternatively expressed isoforms: elevated expression of shorter isoforms lacking the nuclear speckle‐targeting domain shift SRp55 interactions toward cytoplasmic tau pre‐mRNA, explaining why the same kinase that drives tau phosphorylation also drives tau isoform imbalance through an independent subcellular mechanism (Yin et al. 2012; Liu et al. 2008; Yin et al. 2017).

#### 5.2.2 PD: α‐Synucleinopathy and Mitochondrial Links

PD involves abnormal accumulation of α‐synuclein (α‐syn) into intracellular Lewy bodies. Elevated DYRK1A activity promotes this pathological process through multiple mechanisms.

##### 5.2.2.1 Aggregation, Scaffolding, and E3 Ligase Inhibition

One key pathway involves the direct phosphorylation of α‐synuclein at serine 129, a modification that enhances its propensity to aggregate and form Lewy bodies, the hallmark inclusions in PD. (Yong et al. 2023). Additionally, DYRK1A negatively modulates the activity of the E3 ubiquitin ligase Parkin (PRKN) by phosphorylating it at serine 131, thereby inhibiting its ability to tag damaged proteins for degradation and compromising its neuroprotective role in dopaminergic neurons (Im and Chung 2015). DYRK1A also phosphorylates Septin (SEPT4), a structural protein that localizes within α‐synuclein inclusions, suggesting the formation of a pathogenic complex involving DYRK1A, SEPT4 and α‐synuclein (Kim et al. 2006). Importantly, pharmacological inhibition of DYRK1A has demonstrated neuroprotective effects in PD models, including reduced neuronal apoptosis, preservation of mitochondrial function and improved motor performance.

**FIGURE 6:**
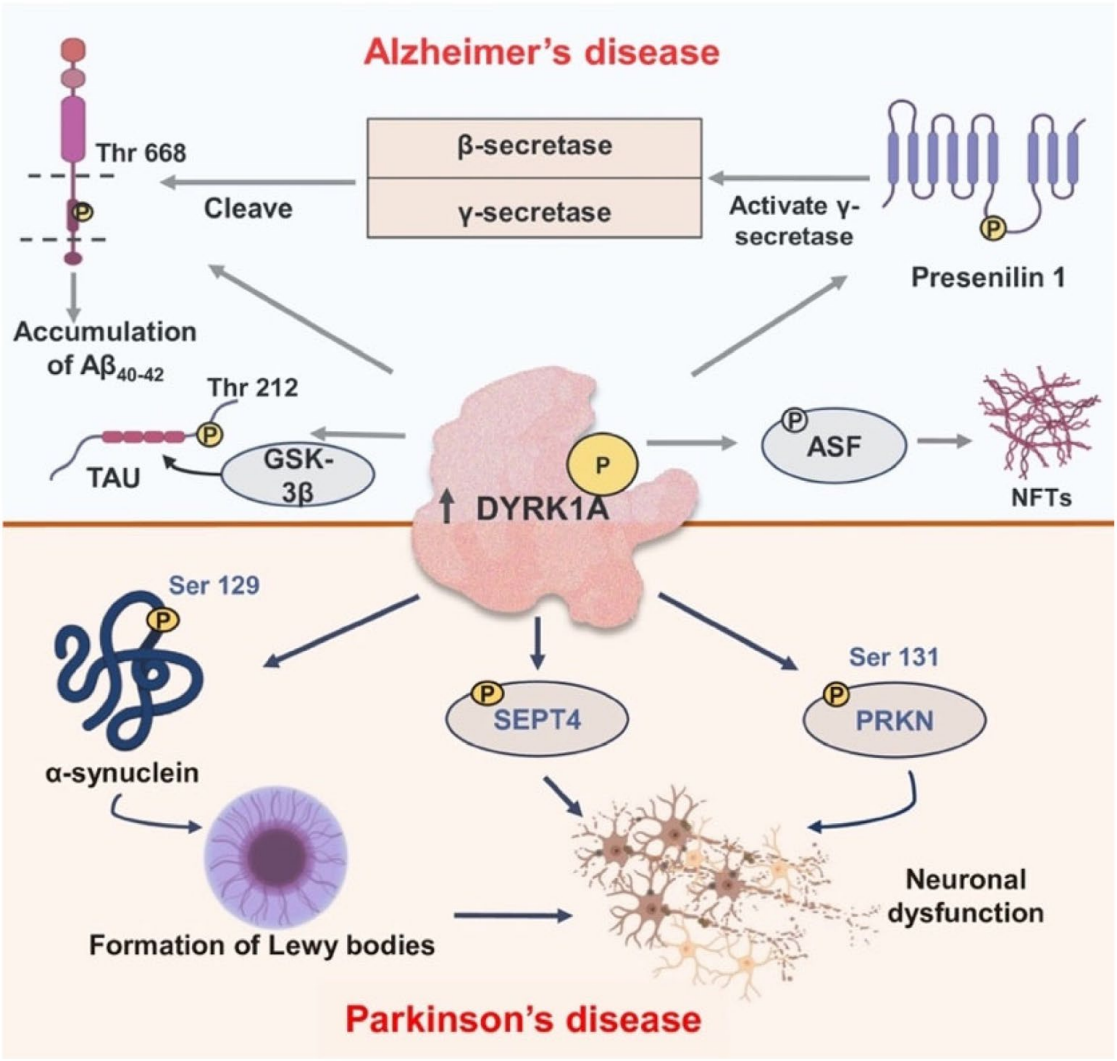
Diagram links elevated DYRK1A to Alzheimer's and Parkinson's pathology: Phosphorylating TAU and α‐synuclein, promoting Aβ accumulation, neurofibrillary tangles, Lewy bodies and consequent neuronal dysfunction, highlighting Thr212, Ser129, Thr668.

These shared benefits across AD and PD reflect a common mechanistic principle. DYRK1A both promotes aggregation‐prone phosphorylation of Tau at Thr212 and α‐synuclein at Ser129 while impairing protein clearance through neprilysin downregulation and Parkin inhibition, forming a pathological feedforward loop that DYRK1A inhibition disrupts (Figure 6) (Cisternas et al. 2025).

#### 5.2.3 Huntington's Disease

In Huntington's disease (HD), elevated expression of DYRK1A phosphorylates Huntingtin Interacting Protein 1 (HIP‐1), exacerbating neuroprogenitor dysfunction. Its inhibition may reduce the aggregation of mutant Huntingtin (HTT) protein, potentially mitigating neurotoxicity and slowing disease progression (Kaltheuner et al. 2021; Kang et al. 2005).

#### 5.2.4 Pick's Disease

In Pick's disease (PiD), DYRK1A shows overactivity throughout the neocortex, hippocampus, and entorhinal cortex. This spatial expression pattern is consistent with the disease neuropathological features, as DYRK1A directly phosphorylates tau protein at threonine 212, a site known to undergo pathological hyperphosphorylation in PiD. This modification contributes to tau aggregation and neuronal dysfunction (Ferrer et al. 2005).

### 5.3 DYRK1A in Cancer and Oncogenesis

**FIGURE 7:**
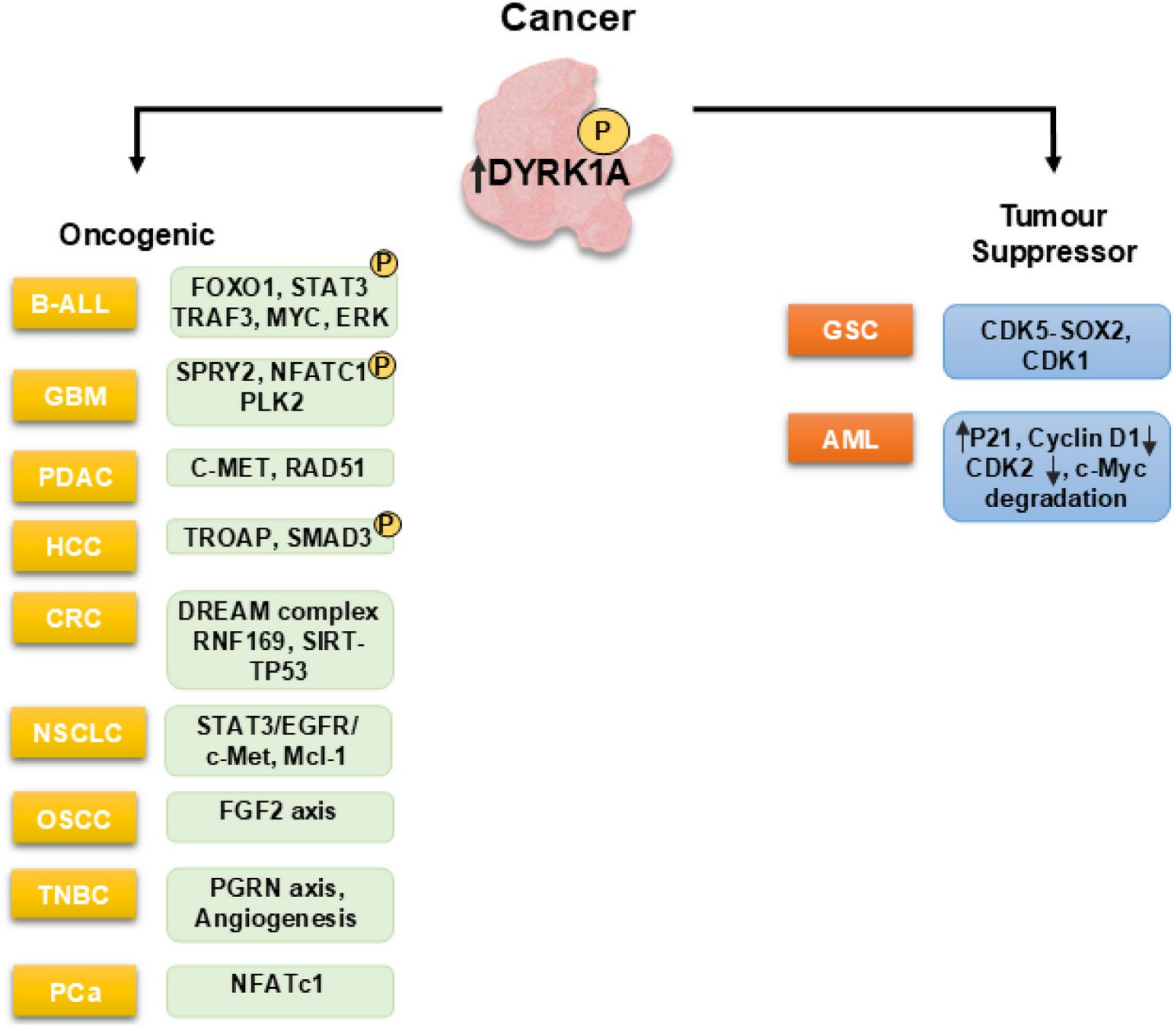
DYRK1A exhibits oncogenic and tumor‐suppressor roles across diverse cancer pathways.

DYRK1A exemplifies a “context‐dependent kinase” whose role in cancer—either oncogenic (tumor‐promoting) or tumor‐suppressive—is strongly dependent on the specific cellular environment, the genetic background of the tumor, and the substrates available. Understanding this duality is critical for developing targeted DYRK1A‐based cancer therapies (Figure 7) (Rammohan et al. 2022).

#### 5.3.1 Hematological Malignancies: Contrasting Roles

DYRK1A exhibits dramatically different roles in acute leukemias, underscoring its dual nature.

##### 5.3.1.1 B‐Cell Acute Lymphoblastic Leukemia

In B‐Cell Acute Lymphoblastic Leukemia (B‐ALL), particularly in cases associated with Down Syndrome‐associated ALL (DS‐ALL) and KMT2A‐rearranged (KMT2A‐R ALL), DYRK1A is overexpressed and functions as an oncogene. The transcription factor KMT2A (a component of leukemia fusion proteins) directly upregulates DYRK1A expression transcriptionally via the essential regulatory unit menin binding to its promoter region. Mechanistically, DYRK1A promotes leukemogenesis through several interconnected pathways: (Ayyadevara et al. 2025).

- DYRK1A regulates B‐ALL via phosphorylation of key transcription factors, FOXO1 and STAT3. DYRK1A phosphorylates and degrades FOXO1, reducing its nuclear entry; inhibiting DYRK1A may therefore increase chemosensitivity (Bhansali et al. 2021).
- DYRK1A activates the BAFF‐induced noncanonical NF‐κB pathway by mediating the phosphorylation of TRAF3, a pathway that promotes both autoimmunity and B‐cell leukemogenesis (Li, Xie, et al. 2021).
- Pharmacological inhibition of DYRK1A unexpectedly results in the hyperphosphorylation of ERK specifically in KMT2A‐R‐ALL, a stress response that induces negative selection and cell cycle arrest (Ayyadevara et al. 2025).

##### 5.3.1.2 Acute Myelogenous Leukemia

In striking contrast to B‐ALL, DYRK1A frequently serves a tumor‐suppressive role in Acute Myelogenous Leukemia (AML), where its mRNA level is often significantly lower than in healthy controls. Upregulation of DYRK1A in AML cells induces G0/G1 cell‐cycle arrest and promotes apoptosis by simultaneously increasing the cell‐cycle inhibitor p21, downregulating the proliferation‐driving proteins Cyclin D1 and CDK2, and promoting *c‐Myc* degradation (Liu, Liu, et al. 2014). These opposing roles reveal that the direction of DYRK1A: tumor‐suppressive in AML versus oncogenic in B‐ALL, which is not intrinsic to the kinase but determined by p53 pathway integrity and the dominant oncogenic transcriptional program of the tumor cell (Ayyadevara et al. 2025).

#### 5.3.2 Solid Tumors: Receptor Stabilization and Resistance

DYRK1A promotes survival and progression across various solid tumor types by modulating stability and resistance mechanisms.

##### 5.3.2.1 Glioblastoma

DYRK1A plays a significant role in Glioblastoma (GBM) by regulating growth factor receptors and stem cell differentiation. Its primary oncogenic interaction involves the epidermal growth factor receptor (EGFR). DYRK1A acts as a stabilizer of EGFR by phosphorylating Sprouty2 (SPRY2) at Thr75. This phosphorylation antagonizes EGFR lysosomal degradation and facilitates its recycling back to the cell surface, thus promoting proliferation. Furthermore, DYRK1A enhances GBM cell migration by activating the transcription factor NFATC1 (Liu, Sun, et al. 2021; Pozo et al. 2013). Conversely, DYRK1A may also act as a tumor suppressor by promoting the differentiation of Glioblastoma Stem Cells (GSCs) via negative regulation of the CDK5‐SOX2 pathway and by negatively regulating CDK1 activity. Recent findings also showed DYRK1A interacts with and phosphorylates PLK2 at Ser358, enhancing PLK2 protein stability and kinase activity, contributing to GBM malignancy (Chen et al. 2021; Tan et al. 2023).

##### 5.3.2.2 Pancreatic Ductal Adenocarcinoma

DYRK1A is highly expressed in Pancreatic Ductal Adenocarcinoma (PDAC), driving tumor growth via *c‐MET* modulation and USP22‐dependent proliferation (Bai et al. 2020). Crucially, DYRK1A is a sensitive target for radiotherapy sensitization. DYRK1A LoF increases DNA Double‐Strand Breaks (DSBs) and impairs Homologous Recombination (HR) by decreasing the key protein RAD51. In EGFR‐amplified tumors such as GBM and PDAC, DYRK1A stabilizes receptor signaling via SPRY1 phosphorylation, while simultaneously reinforcing survival through ATM/ATR and RNF169‐dependent DNA‐repair pathways, explaining why EGFR amplification status, rather than DYRK1A expression alone, determines its pro‐tumorigenic output in solid tumors (Pascual‐Sabater et al. 2025; Lan, Zeng, et al. 2022).

##### 5.3.2.3 Hepatocellular Carcinoma

In hepatocellular carcinoma (HCC), DYRK1A mediates oncogenic function through multiple mechanisms, including stabilization of TROAP (trophinin‐associated protein) (Li, Wei, et al. 2021). Most uniquely, DYRK1A promotes metabolic resistance to OXPHOS inhibition (e.g., IACS‐010759) by directly interacting with and phosphorylating SMAD3 at Thr132. This phosphorylation suppresses the negative impact of TGF‐β signaling and maintains expression of the glutamine transporter SLC1A5, allowing the cancer cells to sustain glutamine metabolism and survive under conditions that would normally be metabolically stressful (Cao et al. 2025).

##### 5.3.2.4 Colorectal Cancer

In colorectal cancer (CRC), DYRK1A is the only DYRK family member that is significantly upregulated in late tumor stages (IIIA to IVB) and is strongly associated with poor prognosis, lower progression‐free survival (PFS), and overall survival (OS). Tumors with high DYRK1A expression are enriched in late metastasis and lymph node stages and exhibit higher tumor recurrence. DYRK1A functionally interacts with the repressive DREAM complex for cell cycle inhibition and the ubiquitin ligase RNF169 for DNA damage response. In microsatellite unstable (MSI) subtypes, high DYRK1A expression is theorized to help cells overcome cellular stress by activating SIRT1, which subsequently inactivates TP53 (Laham et al. 2022).

##### 5.3.2.5 Non‐Small Cell Lung Cancer

DYRK1A is highly expressed in Non‐Small Cell Lung Cancer (NSCLC) tumor samples and is associated with poor overall survival. Inhibition of DYRK1A suppresses *STAT3*/*EGFR*/*c‐Met* signaling. Furthermore, DYRK1A inhibition suppresses expression of the anti‐apoptotic protein *Mcl‐1*, rendering sensitization of NSCLC cells to Bcl‐2 inhibitors (Li et al. 2019).

#### 5.3.3 Other Epithelial Cancers

In oropharyngeal squamous cell carcinoma (OSCC), DYRK1A is critical for maintaining cancer stemness and tumorigenic potential. The DYRK1A‐FGF2 axis regulates this tumor stemness, with inhibition causing a loss of self‐renewal capacity of cancer stem cells (CSCs) (Martin et al. 2021). In prostate cancer (PCa), DYRK1A regulates bone homeostasis by phosphorylating and repressing the osteoclast transcription factor NFATc1; downregulation of DYRK1A via miR‐378a‐3p promotes osteolysis and metastasis (Wang, Du, et al. 2022). In Triple‐Negative Breast Cancer (TNBC), DYRK1A is part of the DYRK1A‐PGRN axis, whose overexpression reverses the EMT (Epithelial‐to‐Mesenchymal Transitions) process inhibited by MiR‐1246. DYRK1A is also associated with angiogenesis in endothelial cells, an essential process for metastasis (Wang, Chen, et al. 2022).

### 5.4 DYRK1A in Metabolic and Cardiovascular Diseases

#### 5.4.1 Diabetes Mellitus: Beta‐Cell Quiescence and Proliferation

DYRK1A is a key player in the pathology of Type 2 Diabetes (T2D), where it negatively regulates pancreatic β‐cell proliferation. Its role is to ensure β‐cell quiescence by preventing cell cycle re‐entry. Here the pathological mode is chronic up‐regulation, not isoform switching. DYRK1A activity is constitutively elevated above the threshold permissive for NFAT nuclear entry, locking β‐cells into a quiescent transcriptional state (Guo et al. 2022).

##### 5.4.1.1 NFAT‐Mediated Repression

DYRK1A tonically suppresses β‐cell proliferation by phosphorylating NFAT transcription factors and forcing their cytoplasmic retention. Accordingly, DYRK1A inhibitors do not merely preserve β‐cell activity, but rather they actively stimulate β‐cell proliferation by restoring nuclear NFAT accumulation and reactivating pro‐proliferative transcriptional programs. Mechanistically, DYRK1A inhibition also disrupts DREAM complex assembly; recent advances show that insulinoma cells proliferate precisely because DREAM complex activity is suppressed (Dirice et al. 2016). Beyond NFAT and DREAM suppression, converging evidence implicates dual‐pathway inhibition as an additional mechanistic amplifier. Natural DYRK1A inhibitors such as harmine and desmethylbellidifolin stimulate β‐cell mass expansion by simultaneously inhibiting DYRK1A‐mediated cytoplasmic NFAT retention and GSK‐3β activity, creating a powerful, convergent pro‐proliferative nuclear signal (Shen et al. 2015; Zheng et al. 2021). The synthetic dual inhibitors GNF4877 and its optimized 6‐azaindole derivative GNF2133 (DYRK1A IC50 = 6.2 nM) recapitulate this convergent mechanism by prolonging NFATc nuclear residency, driving dose‐dependent β‐cell proliferation and improved glycaemic control in vivo, and establishing proof‐of‐concept that combined DYRK1A/GSK‐3β inhibition generates a supra‐additive pro‐proliferative NFAT signal (Liu, Jin, Ding, et al. 2020). Furthermore, DYRK1A inhibitors like leucettines have been shown to additively stimulate insulin production when combined with TGF‐β inhibitors in β‐cell organoids and isolated mouse islets (Pucelik et al. 2023). Clinically, combining DYRK1A inhibitors with GLP‐1 receptor agonists synergistically enhances human β‐cell regeneration rates from approximately 2% (monotherapy) to 5%–6%, accompanied by balance in glucagon level in human islet models, offering a highly translatable, long‐term therapeutic strategy for both Type 1 and Type 2 diabetes (Figure 5A) (Ackeifi et al. 2020).

##### 5.4.1.2 Insulin Signaling in the Brain

DYRK1A regulates Insulin Receptor Substrate‐1 (IRS‐1) and insulin signaling within the brain, connecting its function to metabolic and neurological disorders (Tian et al. 2019). DYRK1A expression is induced epigenetically via the KAT7/HMGN1 signaling axis in AD models, which is hypothesized to ameliorate insulin resistance. Conversely, chronic high insulin exposure can attenuate central insulin signaling via IRS‐1 serine phosphorylation and degradation (Lu et al. 2024).

#### 5.4.2 Cardiovascular Diseases

In addition to congenital heart defects observed in DS patients, DYRK1A is involved in pathological cardiac remodeling in adults.

##### 5.4.2.1 Myocardial Infarction and Heart Failure

DYRK1A is implicated in the regulation of myocardial infarction (MI). DYRK1A promotes the phosphorylation of the Alternative Shearing Factor (ASF), which modulates the alternative shearing of Calcium/Calmodulin‐dependent protein kinase‐IIδ (CaMKIIδ). Activation of this DYRK1A‐ASF‐CaMKIIδ signaling pathway may enhance MI‐induced heart failure (He et al. 2015; Jiang et al. 2026).

##### 5.4.2.2 Cardiac Repair Mechanisms

DYRK1A activity is a crucial negative factor in adult cardiomyocyte cell cycle activation. Inhibition of DYRK1A is considered a potential repair therapy post‐MI. This involves DYRK1A inhibiting the deposition of acetylation markers: knockdown or inhibition decreases phosphorylation levels of WDR82 and KAT6A and concurrently increases the deposition of H3K27ac and H3K4me3 on cell cycle regulatory promoters. This histone modification induces cardiomyocyte cell cycle activation and supports cardiac repair, improving cardiac function (Lan, Chen, et al. 2022).

### 5.5 DYRK1A in Other Disorders and Cellular Processes

DYRK1A contributes to the development of inflammatory microenvironments, a driving force for diseases like Rheumatoid Arthritis and Psoriasis.

*Rheumatoid arthritis* (*RA*): DYRK1A promotes the proliferation, migration, and invasion of fibroblast‐like synovial cells (FLSs) by inhibiting the negative regulator Spry2 and thereby activating the ERK/MAPK signaling pathway (Guo et al. 2018).

*Psoriasis*: DYRK1A increases psoriasis‐like inflammation by mediating the EGFR signaling pathway (Kim et al. 2023).

*HIV*: DYRK1A controls the expression of Cyclin L2, thereby restricting HIV‐1 replication in macrophages. Inhibition of DYRK1A increases Cyclin L2 expression and replication of HIV‐1. DYRK1A has also been associated with the interaction of the adenovirus E1A oncoprotein via the adaptor protein DCAF7 (Glenewinkel et al. 2016). Various pathways in which DYRK1A is involved are depicted in Figure 5.

## 6 DYRK1A Inhibitors

DYRK1A inhibitors classification based on core chemical scaffolds and interaction modes:

- *ATP‐Competitive inhibitors*
  ATP‐competitive inhibitors directly occupy the ATP‐binding pocket in the DFG‐in conformation and constitute the dominant mechanistic class. However, these inhibitors uniformly suffer a 100–350‐fold loss in cellular potency relative to their biochemical IC_50_ values. This attenuation arises because intracellular ATP concentrations (1–10 mM) vastly exceed the Km value of DYRK1A for ATP (approximately 10–15 μM), creating a > 100‐fold concentration disadvantage that forces inhibitors to compete against a thermodynamically overwhelming substrate surplus (Bui et al. 2014). The primary limitation of ATP‐competitive inhibitors is off‐target toxicity arising from CMGC kinase pocket conservation, which each scaffold group below addresses differently (Lindberg, Deau, Arfwedson, et al. 2023).
*Group I: Natural polyphenols*: Flavones and flavonols such as fisetin and kaempferol form dual H‐bonds at Lys188 and Asp307.
*Group II: Planar tricyclic systems*: β‐carboline alkaloids and related indole‐fused system, typified by harmine, form canonical hinge H‐bonds via the β‐carboline pyridine nitrogen and 7‐methoxy group at Lys188 and Leu241 (Adayev et al. 2011).
*Group III: Synthetic azines and diazines*: Synthetic scaffolds incorporating pyrimidine, pyridazine, quinazoline, and imidazo‐pyridazine cores unified by H‐bond interactions at hinge residues Leu241, Glu239, and Lys188 (Lin et al. 2022).
- *Non‐competitive and allosteric inhibitors*
  Non‐competitive and allosteric inhibitors bind outside the ATP pocket or stabilize inactive kinase conformations, providing superior cellular potency retention (10–50‐fold loss versus 100–350‐fold for ATP‐competitive agents) and significantly improved CMGC selectivity. This section encompasses two scaffold families with distinct non‐canonical binding modes (Umezawa and Kii 2021; Liu et al. 2022).
*Group IV: Catechin polyphenols*: EGCG and its fluorinated analogs are examples of non‐competitive inhibitors, which bind to a site distinct from the ATP pocket (Delabar et al. 2024).
*Group V: Sulfur‐containing heterocycles and non‐canonical*/*allosteric modulators*: Encompasses DFG‐out thiadiazines, folding‐intermediate‐selective FINDY and co‐chaperone‐targeting CaNDY, each achieving superior CMGC selectivity through non‐canonical engagement (Kumar et al. 2018; Miyazaki et al. 2022).

## 7 Proteolysis‐Targeting Chimeras

Unlike traditional enzymatic inhibitors, Proteolysis‐Targeting Chimeras (PROTACs) function through targeted protein degradation. They induce selective ubiquitination and proteasomal degradation of DYRK1A via bifunctional molecules that link target‐binding ligands to cereblon E3 ubiquitin ligases (Grigglestone and Yeung 2021). For instance, DYR684 selectively degrades DYRK1A at nanomolar concentrations with preferential selectivity over the functionally distinct paralog DYRK1B, a fully active kinase encoded by a separate gene, not an inactive splice variant. Selectivity arises from differences in ternary complex geometry rather than DYRK1B inactivity. Critically, the high molecular weight of PROTACs (700–1100 Da) substantially limits blood–brain barrier penetration, restricting CNS applicability without dedicated delivery strategies, their primary near‐term applications therefore remain in peripheral oncological and metabolic indications (Wilms et al. 2024; Kuemper et al. 2024).

Most of the DYRK1A inhibitors are small molecules that inhibit the kinase's catalytic activity, restoring dysregulated phosphorylation networks implicated in various neuronal disorders and certain types of cancer. Mechanistic trends and disease related information across the inhibitor classes are compiled in Table 3.

**TABLE 3:** DYRK1A inhibitors and their biochemical mechanism and therapeutic context with disease relevant evidence.

S. no	Derivative	Disease	Mechanism	References
1.	Harmine (β‐carboline alkaloid) 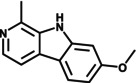	Down Syndrome (DS), Alzheimer's Disease (AD), Diabetes, Neurodegenerative diseases	Acts as an ATP‐competitive DYRK1A inhibitor. Inhibition promotes pancreatic β‐cell proliferation and inhibits DYRK1A‐mediated phosphorylation of substrates like Tau	Kumar et al. (2020); Frost et al. (2011)
2.	AnnH75 (Harmine Derivative) 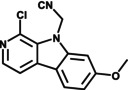	DS, AD, DYRK1A‐associated pathologies	A highly optimized β‐carboline inhibitor designed to eliminate MAO‐A inhibition. It selectively inhibits DYRK1A (IC_50_ 10 nM) and subdues DYRK1A substrates like SF3B1, SEPT4, and tau	Rüben et al. (2015)
3.	Aristolactam BIII (Natural product) 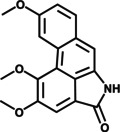	Down Syndrome (DS), DYRK1A‐related diseases	Potent DYRK1A inhibitor (IC_50_ = 9.67 nM in vitro). Suppresses DYRK1A‐mediated hyperphosphorylation of Tau in mammalian cells. Rescues neurological and phenotypic defects in DS‐like models	Choi et al. (2021)
4.	Epigallocatechin‐3‐gallate (EGCG) (Polyphenol) 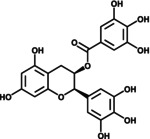	Down Syndrome (DS), Huntington's Disease (HD)	A potent DYRK1A inhibitor characterized as a Class I (non‐ATP competitive) inhibitor. It is known to correct cognitive deficits in DS models and inhibit the aggregation of toxic huntingtin protein in HD	Delabar et al. (2024)
5.	INDY (Inhibitor of DYRK) 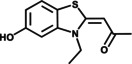	Neurodegenerative diseases, AD	An ATP‐competitive DYRK1A inhibitor (IC_50_ = 240 nM). It binds to the ATP‐binding site and is dual active against DYRK1B and CLK2. Importantly, unlike Harmine, it does not target monoamine oxidase	Ogawa et al. (2010)
6.	Leucettine L41 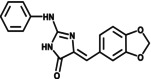	Neurodegenerative diseases, DYRK1A targets	A dual DYRK1A/CLK1 inhibitor (IC_50_ 10–60 nM) that acts in an ATP competitive manner. The carbonyl oxygen of the 2‐aminoimidazolone moiety functions as a hydrogen bond acceptor for Lys188	Naert et al. (2015)
7.	EHT 5372 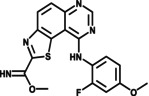	Neurodegenerative disorders	Highly potent DYRK1A inhibitor (IC_50_ 0.22 nM) that inhibits cellular DYRK1A‐mediated tau phosphorylation and Aβ production. Notably exhibits striking selectivity compared to closely related kinases	Coutadeur et al. (2015)
8.	DANDY (Diaryl‐Azaindole Inhibitor) 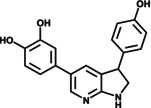	Down syndrome (DS), AD, Glioblastoma	Potent ATP‐competitive inhibitors (Ki values under 12 nM) that function by occupying the ATP binding site. Inhibition has potential therapeutic applications in neurological and oncological disorder	Gourdain et al. (2013)
9.	FINDY 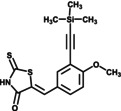	DYRK1A‐associated pathology	Unique inhibitor that targets the DYRK1A folding intermediate by inhibiting Ser97 autophosphorylation, leading to protein degradation. It is a non‐ATP‐competitive inhibitor and does not inhibit mature kinases	Kii et al. (2016)
10.	Lorecivivint 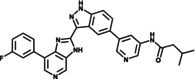	Knee osteoarthritis	Modulates the Wnt pathway by inhibiting two target kinases: CLK2 and DYRK1A	Deshmukh et al. (2019)
11.	GNF2133 (6‐azaindole derivative) 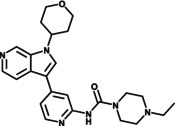	Type 1 diabetes	Potent DYRK1A inhibitor (IC_50_ = 6.2 nM) that selectively inhibits DYRK1A and GSK3β, acting synergistically to promote β‐cell proliferation. Activates NFAT signaling by increasing its residential time in the nucleus	Liu, Jin, Zou, et al. (2020)
12.	KuFal194 (10‐iodo‐11H‐indolo[3,2‐c] quinoline‐6‐carboxylic acid) 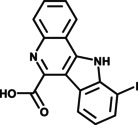	Neurodegenerative disorders	Highly potent in vitro DYRK1A inhibitor (IC_50_ = 6 nM). It is highly selective compared to DYRK1B and CLK1 and inhibits DYRK1A‐mediated tau phosphorylation	Falke et al. (2015)
13.	Meridianins (e.g., N‐morpholinoyl derivative) 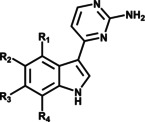	Neurodegenerative diseases, Anti‐proliferative	DYRK1A inhibitors that inhibit various protein kinases. The inhibition mechanism involves ATP‐like interactions attributed to the 2‐aminopyrimidine core	Yadav et al. (2015)
14.	Y020‐3945 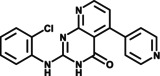	AD, DS	A selective, ATP‐competitive DYRK1A inhibitor (IC_50_ 0.53 μM). It reduces tau phosphorylation, reverses tubulin polymerization, and decreases inflammatory cytokines (IL‐6, TNF‐α)	Wu et al. (2024)
15.	SM07883 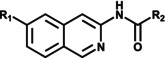	AD, DYRK1A‐associated pathologies	An orally bioavailable DYRK1A inhibitor. It reduces Tau pathology. It shows potent inhibition toward multiple related kinases, including DYRK1B, CLK4, and GSK3β	Melchior et al. (2019)
16.	EHT 1610 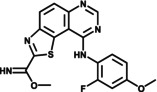	Neurodegenerative disorders, Cancer	Highly potent DYRK1A inhibitor (IC_50_ 0.36 nM). It inhibits kinase activity by competing with ATP. Its binding model is unusual, possibly leading to high selectivity	Jin et al. (2023)
17.	KTD‐092 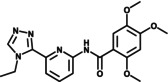	AD, neurodegenerative diseases	An ATP‐competitive inhibitor. It reduces Tau phosphorylation in vitro. The mode of inhibition involves strong hydrogen bonds and hydrophobic interactions in the ATP binding site	Hong et al. (2025)
18.	4‐chlorocyclohepta[b] indol‐10(5H)‐one (Compound 11) 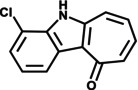	DYRK1A‐associated pathologies	Identified as a submicromolar dual DYRK1A/CLK1 inhibitor. Analysis confirmed a typical Type‐I binding mode, primarily utilizing shape complementarity for tight binding	Lechner et al. (2019)
19.	Acrifoline 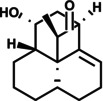	DYRK1A‐associated pathologies	An acridone alkaloid demonstrating potent inhibition against DYRK1A in vitro. It is a naturally occurring DYRK1A inhibitor	Beniddir et al. (2014)
20.	CaNDY (CANDY) 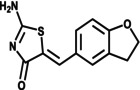	Neurodegenerative diseases, Cancer	Targets the CDC37‐HSP90 co‐chaperone complex. It is an ATP‐competitive inhibitor (IC_50_ 7.9 nM) that restricts DYRK1A maturation, leading to a reduction in active DYRK1A molecules in cells	Sonamoto et al. (2015)
21.	FRTX‐02 (VRN024219) 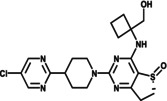	Autoimmune conditions	Functions as a highly potent DYRK1 inhibitor (IC_50_ 2.9 nM for DYRK1A) by competing with ATP. Exhibits dual inhibition profile against multiple CMGC kinases (DYRK1A, DYRK1B, CLK1, CLK2)	Kim et al. (2023)
22.	Di‐Fluorinated Catechin Derivative (Compound 1 h) 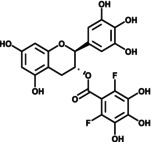	DS, AD	A highly optimized polyphenol derivative acting as a potent ATP competitive DYRK1A inhibitor. Designed to overcome the poor pharmacokinetics of the natural product EGCG	Araldi and Hwang (2022)
23.	Dihydroquinoline derivatives (Compound 1 h) 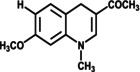	AD	Novel class of dual DYRK1A/CLK1 inhibitors that bind in an ATP‐competitive manner (“INDY way”). They also possess potential antioxidant activity to combat oxidative stress associated with AD hallmarks	Ţînţaş et al. (2022)
24.	Chromeno[3,4‐b] indoles 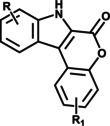	Neurological or oncological disorders	Inhibits DYRK1A kinase activity (IC_50_ 74 nM and 76 nM for the leads) via an ATP active site interaction similar to Lamellarin. Developed as a selective inhibitor with preserved selectivity against CDK and GSK3	Neagoie et al. (2012)
25.	Indenone derivative 4 k 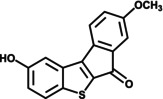	DYRK1A‐associated pathologies	Potent ATP‐competitive DYRK1A inhibitor (IC_50_ = 35 nM). The rigidified structure improves inhibition by binding tightly to the ATP active site	Faouzi et al. (2025)
26.	Leucettinib‐21 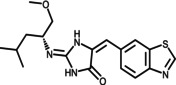	AD, DS, Glioblastoma	It inhibits DYRK1A kinase activity, reduces phosphorylation of Thr286‐cyclin D1 and Thr212‐Tau, thereby correcting cognitive deficits.	Lindberg, Deau, Miege, et al. (2023)

## 8 Therapeutic Approaches for DYRK1A Haploinsufficiency

Unlike DS, where excess DYRK1A activity is the therapeutic target, haploinsufficiency requires the opposite: strategies that restore functional DYRK1A protein to the dosage threshold necessary for normal neuronal development. Three approaches are currently under investigation, each acting at a different biological level.

### 8.1 Gene Replacement via AAV Vectors

Adeno‐associated virus‐mediated delivery of full‐length DYRK1A cDNA (~1.2 kb, well within AAV packaging capacity) offers the most direct route to protein restoration. Serotype selection critically determines CNS coverage: AAV9 and AAVrh10 cross the blood brain barrier (BBB) after systemic neonatal injection and achieve broad cortical and hippocampal transduction, while intracerebroventricular delivery of AAVphp.eB achieves higher CNS transduction efficiency with reduced peripheral expression (Chatterjee et al. 2022; Mathiesen et al. 2020). Proof‐of‐concept for this strategy exists in the analogous haploinsufficiency disorders MECP2 deficiency (Rett syndrome) and CDKL5 deficiency, both of which have shown functional rescue in preclinical AAV models. The key limitations are pre‐existing anti‐capsid neutralizing antibodies that reduce transduction efficiency, the narrow neonatal developmental window within which gene replacement is most effective, and the risk of supraphysiological DYRK1A expression if vector dose is not precisely calibrated. A concern unique to dosage‐sensitive kinases where both excess and deficiency are independently pathogenic. No DYRK1A‐specific AAV trial has been initiated to date (Voronin et al. 2024; Ortiz‐Abalia et al. 2008).

### 8.2 Neurotrophic Factor Augmentation

DYRK1A haploinsufficiency reduces mTORC1 activity by impairing TSC2 phosphorylation at Thr1462, resulting in decreased neuronal cell size and cortical mass. In *Dyrk1a+*/*−* mice, reduced phosphor‐S6K and phosphor‐S6 levels confirm mTOR hypoactivation as the proximal cause of microcephaly. Two interventions have rescued this phenotype in neonatal mice: genetic suppression of *Pten*, which relieves the PI3K/Akt/mTOR brake and fully restores cortical mass and layer V neuron soma size, and daily subcutaneous injection of (1–3) IGF‐1 (GPE) from postnatal Days 0–7, which rescues cortical mass and dendritic complexity without affecting wild‐type littermates. Importantly, recombinant IGF‐1 (Increlex) is already FDA‐approved for severe primary IGF‐1 deficiency, providing a potential regulatory pathway for repurposing. However, treatment initiated after postnatal Day 7 was ineffective in mice, indicating that the therapeutic window closes with the end of active cortical neurogenesis, the primary translational barrier for this approach (Levy et al. 2021).

### 8.3 CRISPR‐Based Gene Editing

DYRK1A haploinsufficiency arises from three classes of pathogenic variants: nonsense and frameshift mutations, splice‐site variants, and missense mutations near the ATP‐binding site. Base editing (Cytosine base editor [CBE] and Adenine base editor [ABE]) and prime editing can correct each class respectively, without requiring double‐strand breaks, reducing the risk of unintended genomic mutations compared to classical Cas9 nuclease approaches. In iPSC‐derived neuronal models, correction of the pathogenic allele normalizes neural progenitor proliferation, restores dendritic arborization, and recovers downstream substrate phosphorylation including LIN52‐Ser28 and Cyclin D1‐Thr286. The primary translational barriers are: efficient in vivo CNS delivery, as lipid nanoparticles show promise but require further optimization for neuronal tropism; mosaic correction, where partial editing below the therapeutic threshold may be insufficient; and off‐target editing requiring whole‐genome sequencing validation before clinical progression (Najas et al. 2015; Raveau et al. 2018).

## 9 Structural‐Activity Relationship of Dyrk1a Inhibitors

### 9.1 Polyphenol DYRK1A Inhibitors

**FIGURE 8:**
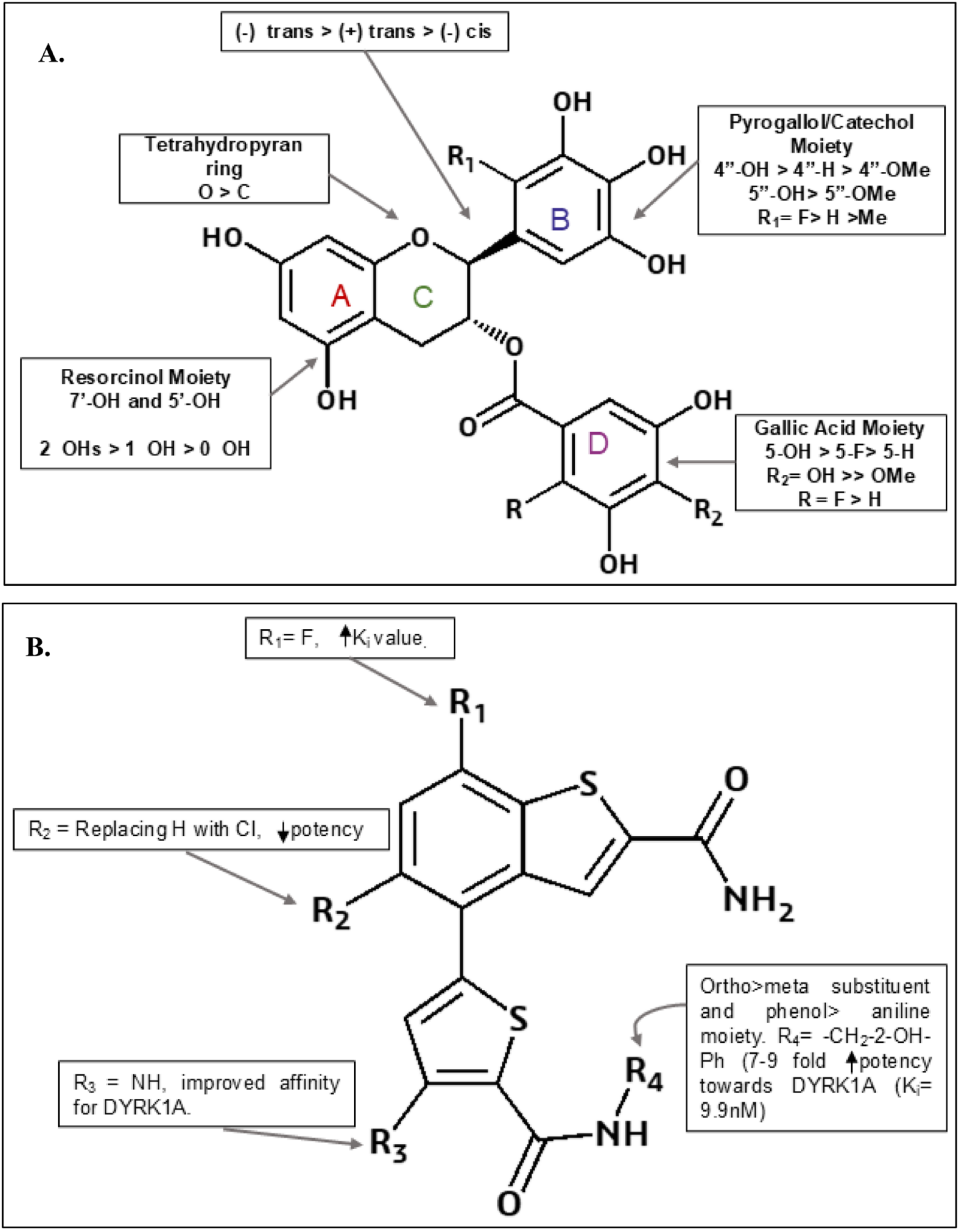
(A) SAR of polyphenol derivative. (B) SAR of Benzothiophene derivative. Created using Chemdraw and Microsoft Powerpoint.

Catechin optimization reveals that trans‐stereochemistry critically governs allosteric pocket complementarity over cis‐isomers. Strategically positioned hydroxyl groups that strongly influence potency: para‐hydroxyl elimination causes a marked loss of activity (EGCG‐4Ome: IC_50_ 25,086 nM vs. EGCG: IC_50_ 232 nM), while meta‐position repositioning reduces potency. Fluorine substitution notably enhances potency beyond classical bioisosteric expectations (1f: IC_50_ 35 nM vs. GCG: IC_50_ 121 nM), suggesting electronically modulating allosteric pocket stabilization and lipophilic complementarity (Araldi and Hwang 2023). Critically, EGCG operates via a non‐ATP‐competitive mechanism confirmed by ATP independence up to 0.8 mM and by the K465R mutation converting inhibition to ATP competitive, establishing this scaffold as strategically superior for CNS indications where intracellular ATP competition reduces cellular potency 100–350‐fold in hinge‐binding chemotypes (Figure 8A) (Delabar et al. 2024; Araldi and Hwang 2022).

### 9.2 Benzothiophene Derivative Inhibitors

Benzothiophene inhibitors display a striking in vitro/cellular potency disconnect revealed only by NanoBRET assay: compound 3j (Ki 9.9 nM in vitro) shows weak cellular binding (IC50 4.36 μM), while aniline derivative 3n achieves superior cellular engagement (IC_50_ 1.1 μM DYRK1A, 0.8 μM DYRK1B) despite lower in vitro potency (Ki 67.8 nM). The data demonstrate that membrane permeability, not affinity, governs therapeutic utility in this scaffold class. In this inhibitor, R_4_ amide substitution revealed nonlinear selectivity patterns: benzene substitution (3c) yielded modest 2.3‐fold potency gains (Ki 56.9–277.9 nM), while phenolic derivatives (3j) achieved 7–9‐fold enhancement (Ki 9.9–65.3 nM) through strategic hydrogen‐bonding at ATP‐site entrances. Critically, R_2_ benzothiophene chlorination showed notable DYRK1B sensitivity (up to 18‐fold potency loss, 3j vs. 3 k) versus minimal DYRK1A impact (8.5‐fold), indicating cryptic mechanistic divergence despite sequence homology (Figure 8B) (Segretti et al. 2022).

### 9.3 β‐Carboline‐Cinnamic Acid

**FIGURE 9:**
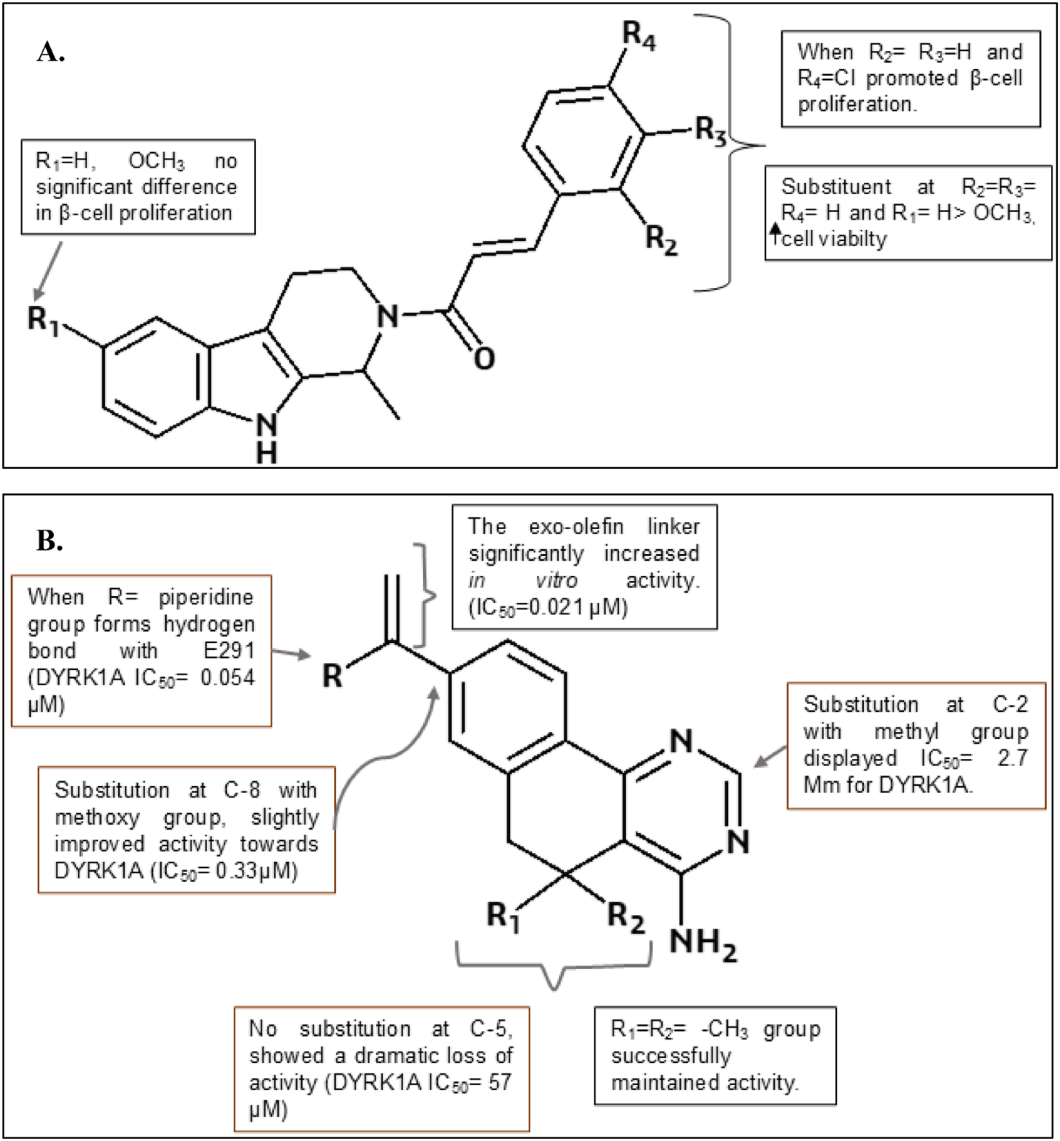
(A) SAR of β‐Carboline‐Cinnamic Acid derivative. (B) SAR of 4‐aminopyrimidine derivative. Created using Chemdraw and Microsoft Powerpoint.

β‐Carboline‐Cinnamic Acid hybrids are mechanistically notable as the only DYRK1A scaffold combining ATP‐competition kinase inhibition with direct β‐cell proliferative activity: most R_1_ = OCH_3_ derivatives stimulate > 210.3% β‐cell proliferation relative to baseline, an effect inseparable from deep ATP‐pocket insertion of the outperforms the β‐carboline skeleton alone (210.3%), consistent with docking results showing deep insertion of the β‐carboline core confirmed by docking. The SAR indicates that para‐chloro substitution on the cinnamamide ring (R_2_ = R_3_ = H, R_4_ = Cl, A_4_, B_4_) is optimal, likely by increasing lipophilicity and complementarity with the hydrophobic back pocket, leading to enhanced β‐cell proliferation. In this context, variation at R1 (H vs. OCH_3_) has little impact, whereas in the unsubstituted series (R_2_ = R_3_ = R_4_ = H) the parent β‐carboline A_1_ (R_1_ = H) clearly surpasses its 6‐methoxy analogue B_1_. (Figure 9A) (Guan et al. 2024).

### 9.4 4‐Aminopyrimidine Derivative

The 4‐aminopyrimidine scaffold emerged as a critical pharmacophoric element for DYRK1A inhibition through systematic ATP‐competitive binding mode analysis. The primary amine at the 4‐position maintains direct hydrogen‐bonding interactions with conserved hinge region residues, a feature essential for kinase inhibitor potency across the CMGC family. 5‐position dimethyl substitution retained activity with improved synthetic tractability. The piperidine moiety at the 8‐position enhanced activity via basicity‐dependent interactions with the catalytic lysine residue. The exo‐olefin linker conversion from the ether oxygen represented a pivotal structural modification that increased activity 10‐fold by improving complementarity with the P‐loop and enabling predicted H‐bonding with Glu291 (modeling derived, not yet crystallographically validated) (Figure 9B) (Fukuda et al. 2018). Comparative kinome profiling across 56 DYRK/CLK inhibitors has confirmed that aminopyrimidine‐class agents exhibit significant CLK1 co‐inhibition at equipotent DYRK1A concentrations, establishing P‐loop extension toward Glu291 as the key structural lever for achieving DYRK1A/CLK1 selectivity in next‐generation analogues (Lindberg, Deau, Arfwedson, et al. 2023).

### 9.5 Flavonols Derivative

**FIGURE 10:**
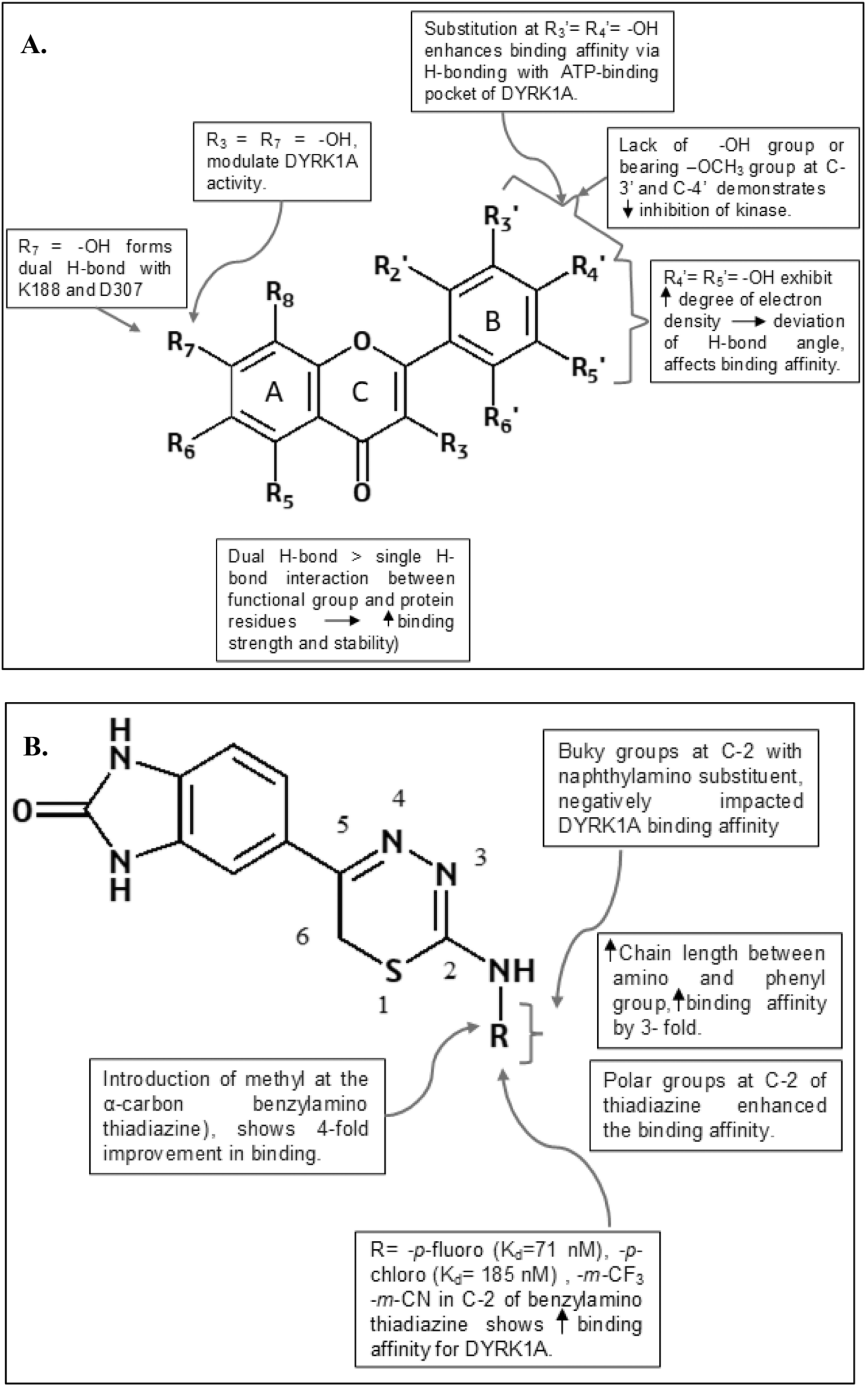
(A) SAR of Flavonols derivative. (B) SAR of Thiadiazine derivative. Created using Chemdraw and Microsoft Powerpoint.

The structure–activity relationship (SAR) analysis of flavonol derivatives targeting DYRK1A elucidates critical scaffolding principles governing kinase inhibition. The twofold potency advantage of fisetin (IC_50_ 149.5 nM) is attributable to the additional Ser242 contact formed by 7‐OH of fisetin at the entrance cleft, a multi‐point engagement gain not achievable by simple B‐ring optimization alone. Conversely, methoxy substitutions (kaempferide) or excessive hydroxylation (myricetin) increase molecular polarity, significantly diminishing enzyme interaction efficacy. The structural requirement for maintained π‐conjugation and planarity is underscored by the complete inactivity of dihydroflavone analogs, establishing that aromatic conjugation is essential for efficient kinase domain penetration. (Figure 10A) (Jin et al. 2025).

### 9.6 Thiadiazine Derivative

The 1,3,4‐thiadiadiazine scaffold exhibits exceptionally sensitive substitution‐dependent binding affinity to DYRK1A, revealing critical structure activity relationships at the 2‐amino position. Systematic introduction of halogenated benzylamino moieties (fluoro‐ and chloro‐) dramatically enhanced DYRK1A binding by 25‐ to 60‐fold, with para‐substituted isomers (3–5: 4‐fluorobenzyl, Kd = 71 nM, 3–2: 4‐chlorobenzyl, Kd = 185 nM) vastly outperforming ortho‐ and meta‐substituted counterparts. Conversely, trifluoromethyl‐ and cyano‐substituted benzylamines exhibited reversed positional preferences (meta > para), indicating distinct steric/electronic requirements for bulkier electron‐withdrawing groups. Chain length modification to phenylethyl (3–22, Kd = 1600 nM) proved tolerable, affording threefold improvement, while naphthylamino substituents negated activity, underscoring steric constraints of the binding site. Importantly, compounds 3–5 from this series induced authentic human β‐cell proliferation at 5 μM. Uniquely among all scaffold families reviewed here, thiadiazines bypass the hinge pharmacophore entirely, making them the only mechanistically Type‐II‐like DYRK1A scaffold and a selectivity‐first design starting point that is orthogonal to every hinge‐anchored chemotype in this section (Figure 10B) (Kumar et al. 2018).

### 9.7 Benzylidene Heterocycles

**FIGURE 11:**
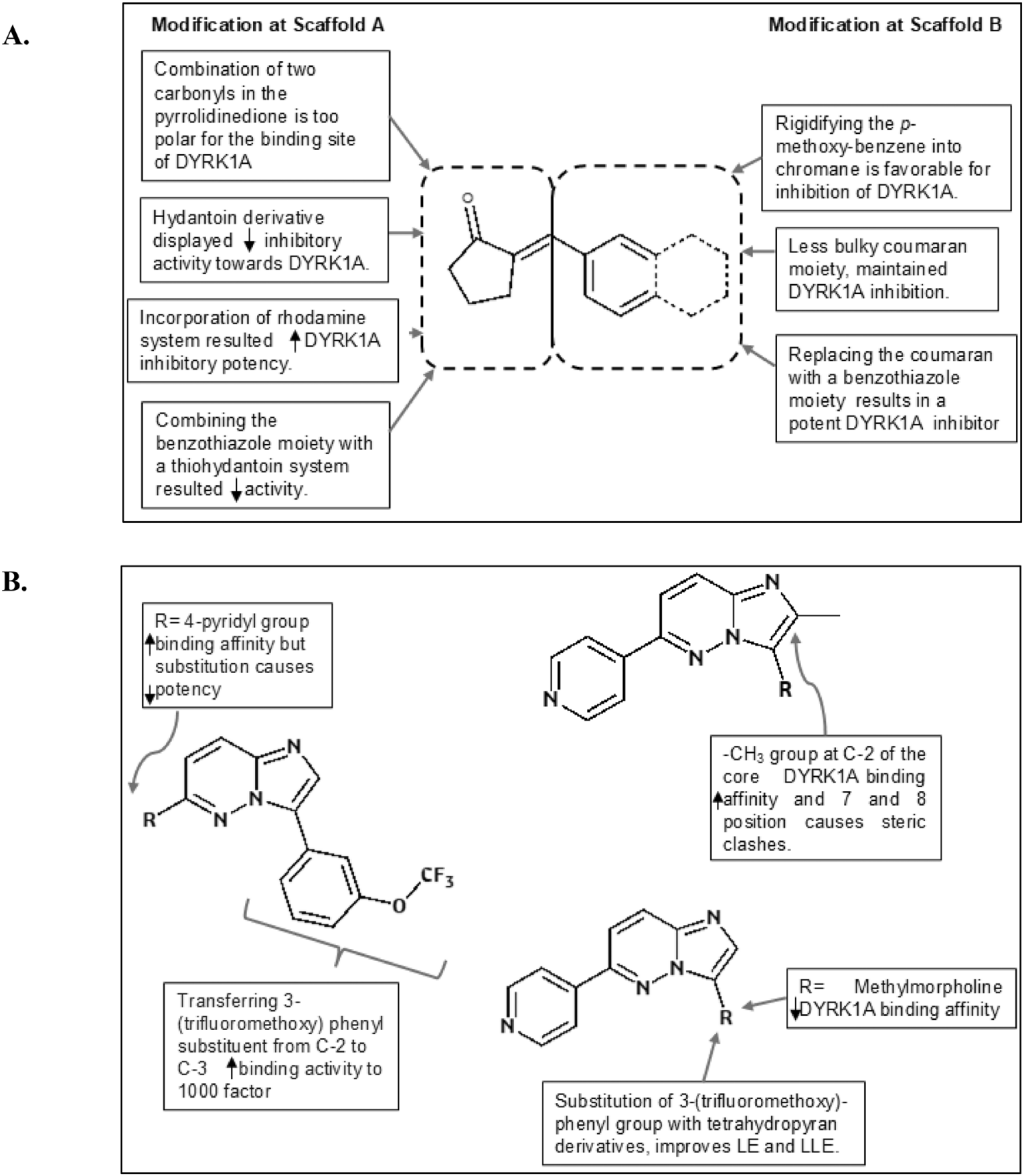
(A) SAR of Benzylidene Heterocycles. (B) SAR of Imidazo[1,2‐b] pyridazines derivative. Created using Chemdraw and Microsoft Powerpoint.

The benzylidene heterocycles prioritized for CNS penetration (molecular weight < 280 g/mol) form Leu241 H‐bonds via their lactone carbonyl. Incorporation of a thione sulfur within the rhodamine core (2‐thioxothiazolidin‐4‐one) acts as a dual hydrogen‐bond acceptor/donor, generating IC_50_ 0.25 μM. However, strategic fusion of bicyclic aromatic systems (benzothiazole or quinoline) to the rhodamine scaffold substantially increases potency, achieving subnanomolar IC_50_ values (down to 0.02 μM). The potency gain results from enhanced shape complementarity within the binding pocket and coordinated interaction with a consensus water molecule at the hinge. Nevertheless, this bicyclic elaboration systematically reduces selectivity toward related CMGC kinases (CDK2, GSK3β, CLK isoforms), reflecting steric encroachment into shared binding regions. Modern selective inhibitors such as pyrazolo [1,5‐b] pyridazine analogues maintain water‐mediated hydrogen‐bonding networks while occupying a shallow lipophilic pocket exclusive to DYRK/CLK kinases, achieving > 100‐fold selectivity over DYRK1B and > 20‐fold over related kinases (Figure 11A) (Mariano et al. 2016). The Leucettinib sub‐series (N2‐substituted 2‐aminoimidazolin‐4‐ones with a benzothiazole‐6‐ylmethylene arm) represents a structural evolution of the leucettamine/benzylidene chemotype, reaching sub‐nanomolar DYRK1A potency (IC_50_ 0.5–20 nM). Regioisomeric iso‐Leucettinibs (IC_50_ > 3–10 μM on DYRK1A), produced by simple positional rearrangement, serve as validated kinase‐inactive negative controls for cellular experiments, a chemogenomic tool unavailable for most other DYRK1A scaffold classes and essential for distinguishing on‐target from off‐target cellular effects (Deau et al. 2023).

### 9.8 Imidazo[1,2‐b] Pyridazines Derivative

Imidazo[1,2‐b] pyridazines represent a well‐characterised DYRK1A chemotype in which activity is governed by substitution around the bicyclic core. Relocation of the lipophilic 3‐trifluoromethoxy phenyl substituent from C‐2 to C‐3 optimally occupies the hydrophobic pocket beneath the glycine‐rich P‐loop, affording a 1000‐fold gain in affinity and improved ligand efficiency. In contrast, steric encroachment from 7‐ or 8‐methyl groups toward the hinge severely disrupts binding. Introduction of a small 2‐methyl substituent is accommodated within the DYRK1A‐specific selectivity cleft, preserving potency while attenuating CLK1 binding. Notably, partial replacement of the 3‐aryl group with more saturated heterocycles can improve solubility with only modest loss of DYRK1A affinity, highlighting this position for ADME‐driven optimization. Subsequent structure‐guided design of compound 29, informed by the X‐ray structure of compound 17, achieved improved CLK selectivity by exploiting a DYRK1A‐specific cavity adjacent to the 2‐methyl group, providing crystallographic confirmation that 2‐Me is the primary single‐atom DYRK1A/CLK1 discrimination handle in this chemotype (Figure 11B) (Henderson et al. 2024).

### 9.9 Chromeno[3,4‐b] Indoles Derivative

**FIGURE 12:**
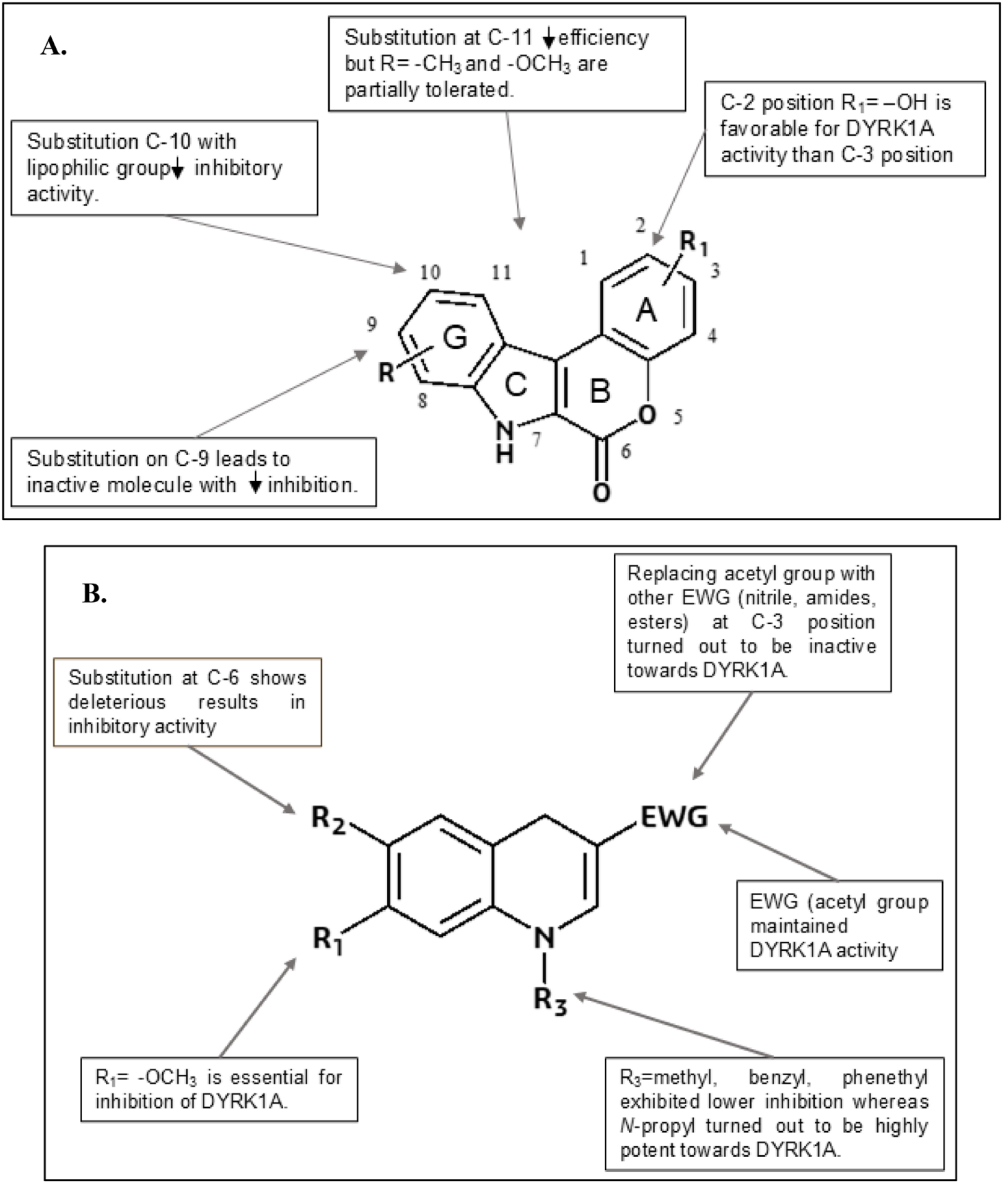
(A) SAR of Chromeno[3,4‐b] indoles derivative. (B) SAR of Dihydroquinolines derivative. Created using Chemdraw and Microsoft Powerpoint.

The potency and selectivity of chromeno[3,4‐b] indole derivatives toward DYRK1A are fundamentally driven by strategic substitutions on phenyl rings A and G, which optimize critical hydrogen‐bonding networks within the kinase ATP‐binding pocket. Hydroxyl groups on ring A are essential for kinase inhibition, functioning as H‐bond donors essential for inhibition engagement. The contacts with Glu239 carbonyl and Leu241 backbone NH at the hinge and catalytic Asp307 and Ser242 are each required. Loss of any single hydroxyl measurably reduces potency. Position C‐2 on ring A demonstrates superior activity and selectivity compared to C‐3, likely due to favorable orientation within the narrow ATP‐binding pocket and minimal steric clashes with the gatekeeper residue. Ring G substitutions at the C‐10 position confer optimal binding, whereas modifications at C‐9 generate substantial steric interference, explaining the observed loss of activity. C‐11 substitutions similarly decrease potency through disruption of the inhibitor backbone‐hinge interaction geometry. The lead compound exhibiting 67 nM IC_50_ reflects cumulative optimization of these interaction points, demonstrating that DYRK1A selectivity arises from precise scaffold positioning and multi‐point engagement with the kinase active site. (Figure 12A) (Neagoie et al. 2012).

### 9.10 Dihydroquinolines Derivative

Structural requirements for hDYRK1 inhibition reflect ATP binding pocket architecture established by INDY/TG003 precedents (PDB 3ANQ). The C‐7 methoxy oxygen establishes a critical hydrogen bond with gatekeeper Leu241 (backbone NH, 2.1Å distance), while the C‐3 acetyl carbonyl bridges the catalytic Lys188 (side chain NH_3_^+^). This dual‐point recognition requires precise 8.5–8.7 Å spacing between oxygens. Only compounds bearing COCH_3_ (C‐3) + OCH_3_ (C‐7) exhibited potency (1 h, 385 ± 130 nM), whereas C‐6 substitutions abolished activity by disrupting geometric alignment. Nitriles, esters, and amides at C‐3 proved inactive, confirming acetyl geometry is non‐negotiable. The 1,4‐dihydroquinoline enamine core is prerequisite; both oxidized quinolinium (2 h) and aromatic quinoline (6 h) were inactive (IC_50_ > 10 μM). N‐alkyl substitution provides secondary optimization: the n‐propyl derivative (1p IC_50_ = 153 ± 44 nM) achieved 2.5‐fold potency gain over n‐methyl (1 h) by optimizing kinase hinge‐region hydrophobic interactions (Figure 12B) (Ţînţaş et al. 2022).

### 9.11 6‐Arylquinazolin‐4‐Amines Derivative

**FIGURE 13:**
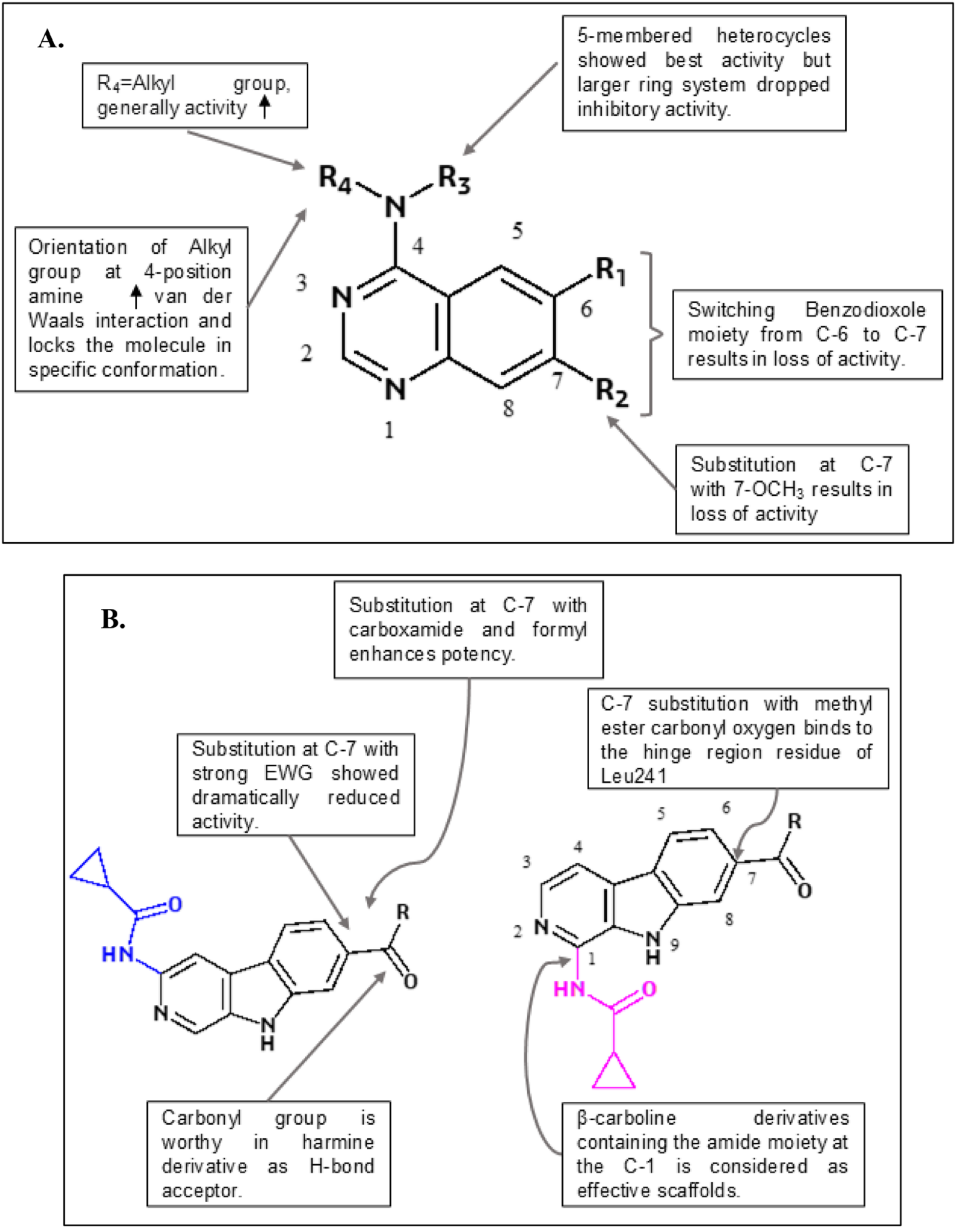
(A) SAR of 6‐arylquinazolin‐4‐amines derivative. (B) SAR of Harmine derivative. Created using Chemdraw and Microsoft Powerpoint.

The 6‐arylquinazolin‐4‐amines scaffold targeting DYRK1A presents a highly position‐dependent pharmacophore wherein substitution at the 6‐position critically determines inhibitor efficacy. The benzo[d]dioxole moiety at this position represents the optimal pharmacophoric element (compound 4, IC_50_ 62 nM): hologram QSAR models confirm it achieves superior affinity via van der Waals contacts with Val173 and Leu294 in the hydrophobic back‐cleft, while the 4‐amino group anchors the scaffold via dual H‐bonds to Lys188 and Leu241 (Leal et al. 2015; Pan et al. 2013). A two‐point recognition motif confers strict positional selectivity at the 6‐position. Displacement of benzo[d]dioxole with isosteric replacements (phenyl, furan) or relocation to the 7‐position abolishes activity entirely (> 10,000 nM), reflecting the precise geometric requirements of the DYRK1A binding pocket and the irreplaceable role of positional specificity in substrate recognition. Conversely, at the 4‐position, alkyl substitution (methyl/ethyl) of the amine linker accommodates a localized hydrophobic binding pocket, enhancing potency through favorable van der Waals interactions (Figure 13A) (Khamouli et al. 2022).

### 9.12 Harmine Derivative

Harmine is a prototypical ATP‐competitive DYRK1A inhibitor whose SAR has been systematically optimized at the 1‐methyl, 7‐methoxy, and 9‐N positions to modulate potency and kinase selectivity. Building on this template, Liu et al. introduced a 1‐cyclopropanecarboxamide and varied the 7‐substituent to tune dual GSK‐3β/DYRK1A activity. In this series, maintaining the harmine‐like β‐carboline core preserved low‐nanomolar DYRK1A inhibition, whereas introducing a 7‐methyl ester as an additional H‐bond acceptor modestly improved DYRK1A potency (IC_50_ 103 nM) relative to the 7‐methoxy analogue (IC_50_ 126 nM) while markedly enhancing GSK‐3β inhibition. Docking shows the 7‐ester carbonyl recapitulates the Leu241 hinge interaction, confirming that fine‐tuning the 7‐position H‐bond acceptor is a powerful handle for dual‐kinase optimization. Among β‐carboline analogues, harmol (7‐OH free, N‐demethylated harmine) achieves comparable DYRK1A potency while showing > 8‐fold reduced MAO‐A inhibition, establishing harmol as a higher‐therapeutic‐window lead than harmine itself and validating the MAO‐A selectivity design rule in Table 4 with an experimentally confirmed analogue (Figure 13B) (Liu, Liu, et al. 2021; Benny et al. 2023).

**TABLE 4:** SAR‐derived design guidelines for 13 DYRK1A inhibitor scaffold classes, organized by binding mechanism, kinase region targeted, inhibitor fragments‐residue interactions, and actionable medicinal chemistry optimization rules for next‐generation analogue design.

S. no.	Scaffold class (inhibitor type)	Kinase region targeted	Inhibitor fragments with interacting residue	Design strategy	References
1.	Polyphenol/Catechin derivatives (non‐ATP‐competitive, allosteric)	Allosteric site (K465, separate from ATP pocket)	*B‐ring para‐OH*: allosteric pocket (H‐bond) *Gallate D‐ring*: K465 locus (hydrophobic, key anchor) *Trans‐OH* (*C2*/*C3*): allosteric cavity walls (conformational fit)	Retaining para‐OH on B‐ring, any methylation abolishes activity. Prioritizing trans‐stereochemistry. Fluorine at para‐B‐ring improves potency beyond standard bioisosteres. ATP‐independence makes this scaffold preferred for CNS targets where intracellular ATP is high	Segretti et al. (2022)
2	Benzothiophene derivatives (ATP‐competitive, Type‐I, entrance cleft)	ATP‐site entrance cleft (Tyr243, Asp247, DYRK1A‐selective)	*R*_*4*_ *phenolic‐OH*: Tyr243 (H‐bond) *R*_*4*_ *phenolic‐OH*: Asp247 (H‐bond) *R*_*2*_ *chloro*: entrance cleft hydrophobic pocket (DYRK1A/B selectivity switch)	Phenolic R_4_ substitution is mandatory for hinge‐cleft H‐bonding R_2_‐Cl confers DYRK1A/1B selectivity, optimizes cellular permeability as biochemical affinity alone does not predict cellular potency	Segretti et al. (2022)
3.	Β‐Carboline‐Cinnamic acid hybrids (ATP‐competitive, Type I, deep pocket)	Hinge + back pocket (Lys188, Leu241, Phe238, Val173)	*Β‐Carboline pyridine N1*: Lys188 (H‐bond, primary anchor) *7‐methoxy group*: Leu241(H‐bond, hinge contact) *Cinnamamide aryl ring*: Phe238/Val173 (hydrophobic back pocket) *Para‐Cl on cinnamamide*: hydrophobic back pocket	The pyridine N1‐Lys188 H‐bond is the non‐negotiable anchor, N‐methylation disrupts it. Para‐Cl on the cinnamamide phenyl is optimal for back‐pocket complementarity. C‐7 substitution on Β‐Carboline modulate dual‐kinase (DYRK1A/GSK1Β) selectivity	Guan et al. (2024); Qiu et al. (2024)
4.	4‐Aminopyrimidine derivatives (ATP‐competitive, Type I, P‐loop extended)	Hinge + *P*‐loop (Glu239, Leu241, Lys188, Glu291)	*4‐Amino group*: Glu239/Leu241 (dual H‐bond, primary anchor) *Piperidine* (*C‐8*): Lys188 (electrostatic) *Exo‐olefin linker*: P‐loop (improved fit) *Amino extension*: Glu291 (H‐bond)	Dual H‐bond at hinge (Glu239/Leu241) must be preserved. Replacing ether oxygen with exo‐olefin for 10‐fold potency gain. Installing piperidine at C‐8 for Lys188 engagement. Target Glu291 for DYRK1A/CLK1 selectivity in next‐generation analogues	Fukuda et al. (2018)
5.	Flavonol derivatives (ATP‐competitive, Type I, multi‐point hinge)	ATP hinge region (Lys188, Asp307, Ser242, Glu239, Leu241)	*C‐7‐OH*: Lys188 + Asp307 (H‐bond) B‐ring 3′‐OH: Glu239 (H‐bond donor) B‐ring 4′‐OH: Leu241 (H‐bond donor) *C‐7‐OH*: Ser242 (H‐bond, entrance cleft)	Preserving free‐OH at C‐7, B‐ring 3′ and 4′, methylation of any single OH reduces potency substantially. Maintaining full aromatic planarity dihydroflavone is completely inactive. Adding a Ser242 contact (as in fisetin) is the most accessible single‐step potency gain	Jin et al. (2025)
6.	1,3,4‐Thiadiazine derivatives (Non‐hinge binding, putative DFG‐out‐pocket)	DFG‐out pocket (Phe238 gatekeeper‐adjacent pocket) (non‐classical, Phe238 bulkiness tolerated via DFG model‐guided design)	*2‐Amino benzylamino*: DFG‐out pocket (vdW contacts near Phe238) *Para‐halogen* (*F*/*Cl*): hydrophobic DFG pocket *Thiadiazine core*: activation loop flank (modeling‐predicted)	Targeting the DFG‐out pocket for inherent DYRK1A/B selectivity unavailable to hinge binders. Using para‐halogen (F or Cl) only, ortho/meta isomers lose selectivity. This is the only scaffold class where hinge engagement is unnecessary. Any attempt of adding hinge contacts as it disrupts DFG‐out stabilization	Kumar et al. (2018)
7.	Benzylidene heterocycles (ATP‐competitive, Type I, shallow lipophilic)	Hinge + shallow lipophilic pocket (Leu241, Ala186, Val173, Leu294)	*Lactone carbonyl*: Leu241 (H‐bond, hinge anchor) *Benzylidene aryl ring*: Ala186/Val173/Leu294 (vdW, lipophilic pocket) *Heterocyclic scaffold*: water bridge (bridging H‐bond at hinge)	Retaining the hinge H‐bond (Leu241) via lactone/carbonyl. Water‐mediated bridging is key to selectivity, disrupting it broadens kinase inhibition. Avoiding bicyclic aromatic fusion unless CLK/CDK co‐inhibition is desired, mono‐cyclic benzylidene gives the best DYRK1A selectivity window	Deau et al. (2023)
8.	Imidazo[1,2‐b] pyridazines (ATP‐competitive, Type I, P‐loop focused)	Hinge + *P*‐loop (Lys188, Leu241), Glu239, sub‐pocket near‐2‐Me position	*C‐2 pyridyl N*: Lys188 (H‐bond) *C‐2 pyridyl N*: Leu241/Glu239 (H‐bond) *C‐2 trifluoromethoxyphenyl*: P‐loop hydrophobic pocket (vdW) *2‐Methyl group*: DYRK1A‐specific selectivity cleft (steric fit, CLK1 exclusion)	Placing lipophilic aryl at C‐3 (not C‐2) for 1000‐fold affinity improvement. Retain 2‐Methyl as the CLK1‐selectivity handle, do not substitute with larger groups as hinge steric clashes abolishes activity. P‐loop aryl substitution is the primary avenue for potency optimization	Henderson et al. (2024)
9.	Chromeno[3,4‐b] Indoles (ATP‐competitive, Type I, P‐loop focused)	ATP hinge region (Glu239, Leu241, Asp307, Ser242, gatekeeper Phe238)	*Ring A C‐2‐OH*: Glu239 (H‐bond donor) *Ring A C‐2‐OH*: Leu241 backbone NH (H‐bond donor) *Ring A OH*: Asp307 + Ser242 (H‐bond, catalytic residues) *Ring G*: Phe238 (vdW, minimal steric clash required)	Every ring A hydroxyl contact is essential, loss of any single‐OH is not tolerated. C‐2 substitution is preferred over C‐3 for proper pocket orientation. Limiting ring G substitutions to C‐10, C‐9 and C‐11 modifications introduce steric clashes with the pocket wall	Neagoie et al. (2012)
10.	Dihydroquinoline derivatives (ATP‐competitive, Type I, dual anchors)	Hinge region (Leu241, Lys188) + adjacent hydrophobic flank	C‐7 methoxy ‘O’: Leu241 backbone NH (H‐bond, 2.1 Å) C‐3 acetyl C=O: Lys188 side‐chain NH_3_^+^ (H‐bond) Enamine core: hinge geometry (maintains 8.5–8.7 Å ‘O‐O’ spacing)	Both C‐3 acetyl and C‐7 methoxy are simultaneously required, removal of the either abolishes activity. Maintaining enamine (1,4‐dihydro) oxidation state, any oxidation to quinolinium or aromatic quinoline is inactive. Substitution at C‐6, it disrupts the dual‐anchor geometry	Ţînţaş et al. (2022)
11.	6‐Arylquinazoline‐4‐amines (ATP‐competitive, Type I, dual H‐bond anchor)	Hinge + back cleft (Lys188, Leu241, Val173, Leu294)	*4‐Amino group*: Lys188 (H‐bond) *4‐Amino group*: Leu241 (H‐bond) *Benzo[d]dioxole at C‐6*: Val173/Leu294 (vdW, hydrophobic back cleft) *4‐N alkyl* (*Me*/*Et*): shallow hydrophobic pocket (vdW, potency modulator)	The 4‐ amino dual H‐bond (Lys188/Leu241) is the non‐negotiable anchor. At C‐6, only benzo[d]dioxole is tolerated, any replacement or positional relocation to C‐7 abolishes activity. Small alkyl group (Me/Et) at 4‐N are the only acceptable potency‐tuning modification	Leal et al. (2015)
12.	Harmine/Β‐carboline derivatives (ATP‐competitive, Type I, hinge‐anchored)	Hinge + back pocket (Lys188, Leu241, back pocket hydrophobic wall)	*Β‐carboline pyridine N1*: Lys188 (H‐bond) *7‐methoxy*/*7‐ester*: Leu241 backbone NH (H‐bond, hinge contact) *N9 methyl*: hydrophobic back pocket (vdW, essential for affinity) *C‐7 substituent*: MAO‐A active site (planarity‐dependent, selectivity handle)	N1‐Lys188 H‐bond and N9 methyl back‐pocket contact are both essential, N9‐demethylation causes potency loss. Tuning C‐7 substitution for target selectivity: polar/bulky groups at C‐7 disrupt MAO‐A while retaining DYRK1A binding. The Β‐carboline core is a privileged scaffold requiring minimal modification for initial kinase engagement	Kumar et al. (2020); Tarpley et al. (2021)
13.	Pyridopyrimidinone derivatives (ATP‐competitive, Type I, hinge anchored)	Hinge region (Leu241, Met240 + shallow hydrophobic pocket) (Lys188 not engaged)	*Pyridopyrimidinone C=O*: Leu241 backbone amide (H‐bond) *2′‐Chloroaniline*: Met240 (vdW, key affinity determinant) *Para‐pyridine ‘N’*: electrostatic microenvironment (additional stabilization)	Retaining hinge H‐bond via C=O to Leu241. 2′‐Cl on the aniline is critical for Met240 contact, unsubstituted aniline substantially reduces activity. The primary design priority for next‐generation analogues is installing a Lys188‐engaging group to overcome the current potency ceiling of this scaffold	Wu et al. (2024)

### 9.13 Pyridopyrimidinone Derivative

**FIGURE 14:**
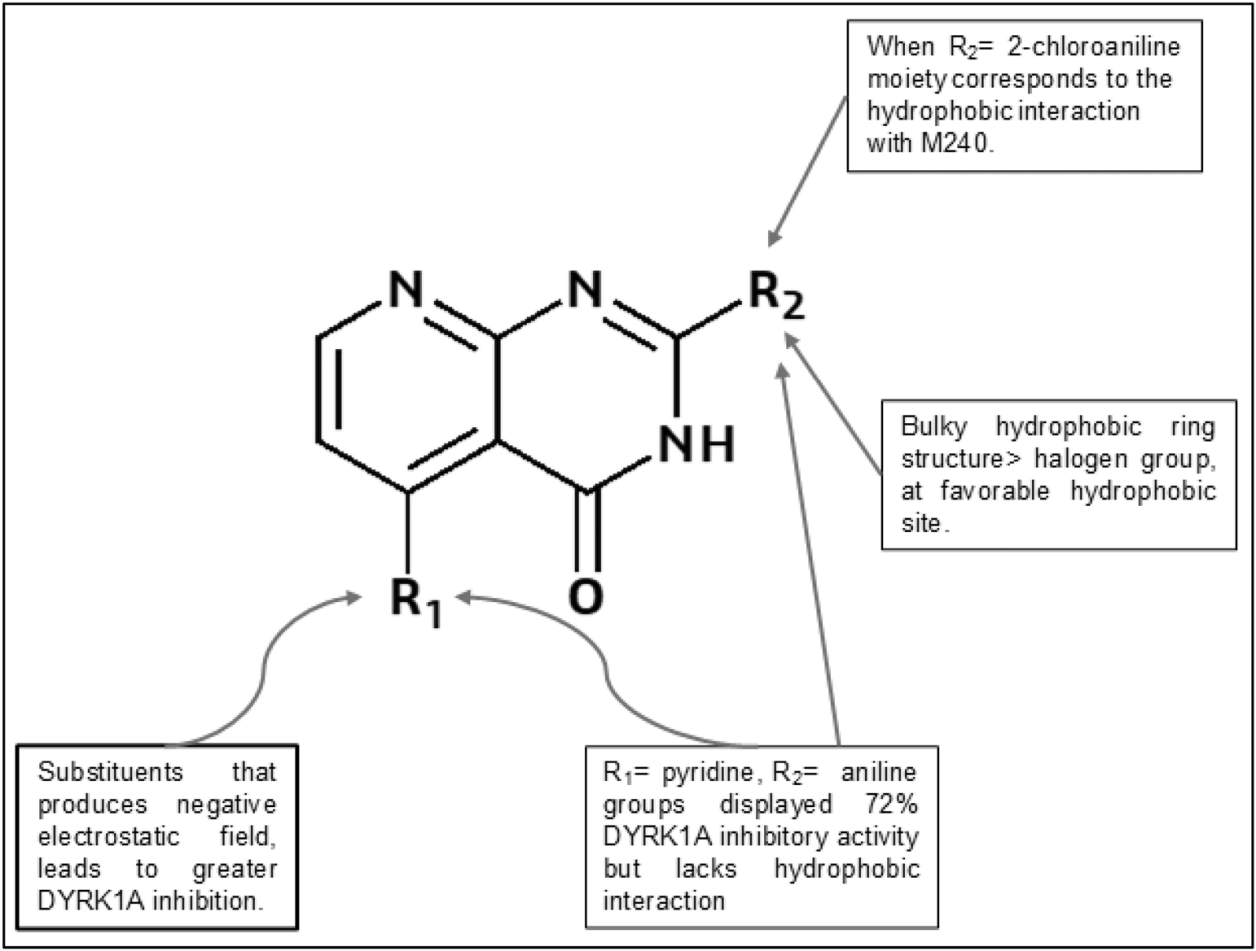
SAR of pyridopyrimidinone derivative. Created using Chemdraw and Microsoft Powerpoint.

The Pyridopyrimidinone scaffold introduces 2‐chloroaniline substitution at the 2′ position extends binding affinity through complementary interactions with the hydrophobic Met240 residue within the ATP‐binding pocket, a site‐specific interaction absent in unsubstituted aniline analogs. The incorporation of halogen atoms (chlorine) at this position is a well‐established medicinal chemistry principle that enhances binding through favorable van der Waals contacts and modulation of lipophilicity within constrained hydrophobic cavities. The para‐positioned pyridine nitrogen occupies a negative electrostatic microenvironment and provides a third binding contact beyond the core hinge H‐bond. Notably, cross‐scaffold profiling of 56 DYRK/CLK inhibitors confirmed that pyridopyrimidinone‐class agents display the narrowest DYRK/CLK selectivity window of all Type I scaffolds tested, consistent with the absence of a Lys188 anchor, making Lys188 engagement the single most actionable structural objective for next‐generation analogues in this series (Figure 14) (Wu et al. 2024).

To facilitate rapid comparison across chemotype classes, the binding region, critical fragment‐residue interactions, and non‐negotiable design rules for each scaffold are consolidated in Table 4. The table is organized by binding mechanism: allosteric non‐ATP‐competitive, ATP‐competitive Type‐I hinge‐anchored, and Type‐II‐like DFG‐out (Yang et al. 2023).

## 10 Emerging Areas of Research: Advanced Formulation Strategies and Future Perspectives

Treatment modalities for DYRK1A have evolved from ATP‐competitive inhibitors to modalities with enhanced specificity, delivery to the CNS, and minimized off‐target effects.

*Proteolysis‐Targeting Chimeras* (*PROTACs*) *and bifunctional degraders*. DYR684 (2024) combine an ATP‐competitive DYRK1A ligand with a CRBN recruiter to engage in a ternary complex leading to K48 polyubiquitination and fast proteasomal degradation. Efficiency of degradation is aided by conformation of kinase‐domain explaining isoform‐specific resistance (Wilms et al. 2024).

*Polypharmacology and multi‐target directed ligands* (*MTDLs*) and combinational therapy (i.e., DYRK1A/GSK3β/FYN, DYRK1A/CLK1) tackle compensatory signaling, synergistically attenuating tau hyperphosphorylation and cognitive impairment in models of Down syndrome (Demuro et al. 2021).

*Molecular glue degraders* (*MGDs*) *and alternative E3 ligase platforms*. MGDs are monovalent small molecules that bring target‐E3 proximity without using linkers, which leads to enhanced permeability and blood–brain barrier (BBB) penetration. Discovery applies HTRF, SPR, high‐content imaging, bioinformatics, and DNA‐encoded libraries to further explore chemical space (Lemaitre et al. 2024; Shi et al. 2021).

*Optogenetics‐based inducible protein degradation systems*, peptide‐mediated OptoTrim‐Away and FLASH‐Away open up the possibility of millisecond, spatially controlled DYRK1A degradation mediated by light‐driven TRIM21 oligomerization. Far‐red actuators and implantable photonic devices facilitate deeper CNS penetration at particular behavioral time windows (Chen et al. 2025; Hu et al. 2024).

*DNA nanotechnology and programmable nanodevices*. Synthetic programmable DNA origami and aptamer‐modified nanostructures carry DYRK1A inhibitors, small interfering RNA (siRNA), or messenger RNA (mRNA) with switchable release mediated by built‐in sensors. Computational modeling and DEL screens facilitate design of CNS‐penetrant degraders and precision delivery vehicles (Kansara et al. 2025; Maïo et al. 2014).

These approaches could be taken forward as research directions for developing DYRK1A‐targeted therapies.

## 11 Summary and Outlook

This review comprehensively maps the multifaceted biological network of DYRK1A, underscoring its dosage‐dependent pathology across diverse human conditions. Rather than merely cataloging compounds, we explored the mechanistic interplay between kinase dysregulation and disease progression, from neurogenesis disruption to tau hyperphosphorylation in cognitive decline, alongside emerging metabolic roles in pancreatic function. Moving forward, realizing the full clinical potential of targeting this enzyme requires a paradigm shift. The historical reliance on traditional orthosteric blockade must evolve. Researchers should prioritize targeted protein degradation frameworks, which offer the unique advantage of eliminating the scaffolding functions of the kinase entirely while sidestepping the severe off‐target liabilities inherent to highly conserved binding pockets. Additionally, integrating advanced biophysical approaches such as surface plasmon resonance and molecular dynamics will be essential to delineate precise ligand‐binding kinetics and rationally design highly selective allosteric modulators. To translate these structural insights in vivo across diverse disease contexts from neurodegeneration to oncology and metabolic disorders, future research must prioritize both optimizing CNS penetrance and developing context‐specific dosing strategies to rigorously manage the inherently dosage‐sensitive nature of DYRK1A. Ultimately, coupling this structurally guided design with the validation of reliable clinical biomarkers and synergistic drug combinations will forge a pragmatic, stepwise path to safely evaluate these transformative therapeutics in complex human trials.

## 12 Conclusion

DYRK1A has emerged as a high‐value yet profoundly complex therapeutic target whose strict gene dosage dictates cellular health. Translating its immense potential into the clinical now hinges on uncoupling its pathogenic activity from essential physiological functions. Mastering this precise molecular balance will be the defining step in advancing from basic kinase biology to safe, disease‐modifying therapies.

## Author Contributions

**Sampriti Paul:** conceptualization, writing – original draft, data curation, formal analysis. **Prashant Tiwari:** writing – review and editing, supervision, validation. **Sonal Dubey:** validation, formal analysis, visualization.

## Funding

The authors have nothing to report.

## Ethics Statement

The authors have nothing to report.

## Conflicts of Interest

The authors declare no conflicts of interest.

## Data Availability

Data sharing not applicable to this article as no datasets were generated or analysed during the current study.
